# Synthesis of 7,2′-Dihydroxy-4′,5′-Dimethoxyisoflavanone, a Phytoestrogen with Derma Papilla Cell Proliferative Activity

**DOI:** 10.3390/molecules27196660

**Published:** 2022-10-07

**Authors:** Taewoo Kim, Hyun Su Kim, Jaebong Jang, Dong-Jun Kim, Jongkook Lee, Dongjoo Lee, Seok-Ho Kim

**Affiliations:** 1 College of Pharmacy and Institute of Pharmaceutical Sciences, CHA University, 120 Haeryong-ro, Pocheon-si, Gyeonggi-do 11160, Korea; 2 College of Pharmacy, Korea University, Sejong 30019, Korea; 3 College of Pharmacy, Kangwon National University, Chuncheon, Gangwon-do 24341, Korea; 4 College of Pharmacy and Research Institute of Pharmaceutical Science and Technology (RIPST), Ajou University, 206 Worldcup-ro, Yeongtong-gu, Suwon 16499, Korea

**Keywords:** phytoestrogen, isoflavanone, *Dalbergia oliveri*, hair growth, deoxybenzoin, total synthesis

## Abstract

This paper reports a concise and scalable method for the synthesis of the phytoestrogen 7,2′-dihydroxy-4′,5′-dimethoxyisoflavanone **1** via an optimized synthetic route. Compound **1** was readily obtained in 11 steps and 11% overall yield on a gram scale from commercially available 3,4-dimethoxyphenol. The key features of the synthesis include the construction of the deoxybenzoin unit through a sequence of Claisen rearrangement, oxidative cleavage, and aryllithium addition and the efficient synthesis of the isoflavanone architecture from highly functionalized 2-hydroxyketone.

## 1. Introduction

Phytoestrogens are naturally occurring dietary compounds. They are found in a wide variety of foods, including fruits, vegetables, and grains [1,2,3,4]. These plant-derived compounds and their metabolites are structurally and functionally similar to those of mammalian estrogens, such as estradiol, and thereby exhibit weak estrogenic and anti-estrogenic effects by binding to estrogen receptors (ERs) [3,5,6]. There is increasing evidence that edible phytoestrogens have numerous health benefits, including anticancer, antioxidant, anti-inflammatory, hepatoprotective, antibacterial, and antiviral activities, that are closely related to the prevention and treatment of various types of cancers, cardiovascular diseases, osteoporosis, neurological diseases, diabetes/obesity, immune system dysfunction, menopause symptoms, and skin aging conditions, such as alopecia [1,2,3,5,7,8]. Hence, phytoestrogens are promising non-steroidal estrogenic compounds and potential alternatives to estrogen replacement therapy (ERT) for human healthcare.

Phytoestrogens are classified into several subgroups, such as flavonoids, isoflavonoids, and lignans, based on their structural motifs and biosynthetic pathways. Furthermore, isoflavonoids vary among subclasses, such as isoflavones, isoflavanones, isoflavans, pterocarpanes, and coumestans [1,2,9,10]. Owing to their structural rarity, as well as their unique and diverse range of biological functions, the chemistry related to the isolation, structural elucidation, and bioactivities of isoflavanones, along with their therapeutic applications, has been extensively investigated [10,11,12].

**Figure 1:**
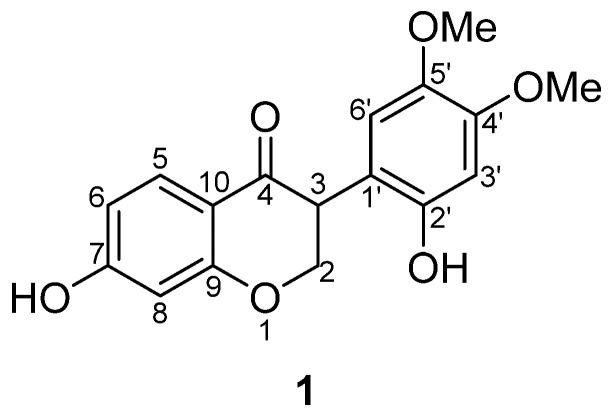
Chemical structure of 7,2′-dihydroxy-4′,5′-dimethoxyisoflavanone (**1**).

In 2003, 7,2′-dihydroxy-4′,5′-dimethoxyisoflavanone (**1**) was first isolated from the heartwood of *Dalbergia louvelii* R. Viguier (Fabaceae), which was used as a folk medicine to treat bilharzia and malaria in Madagascar [13]. More recently, **1** and its structurally related phytochemicals possessing diverse substitution patterns and oxidation states were isolated from the bark of *Dalbergia oliveri* Prain, a traditional Thai medicine used for the treatment of chronic ulcers in Southeast Asia (Figure 1) [14]. Isoflavanone **1** exhibits potent hair growth effects on immortalized dermal papilla cells (iDPCs). In a cell proliferation assay, **1** induced significant cell proliferative activity (54.1% at 10 μM, EC_50_ = 8.83 μM), which was more potent than that of Minoxidil (20.6% at 10 μM), a widely used medication for the prevention and treatment of hair loss [14]. Moreover, isoflavanone **1** induced the anagen phase of the hair cycle in a mouse model via subcutaneous (SC) injection [15].

In 2018, Kim et al. reported a sophisticated approach for the synthesis of **1**. However, this synthetic route does not yield a large amount of isoflavanone **1**, although the synthetic steps are relatively short [15]. Practically, the preparation of naturally occurring compounds in large quantities is a highly formidable task because the large-scale collection and isolation of natural products from natural sources are restricted. Therefore, we have been exploring an efficient and scalable synthetic strategy for the large-scale synthesis of **1** to identify the mechanism of its hair growth effects through *in vivo* animal model studies, as well as its wide range of health benefits. Herein, we report the synthesis of the natural isoflavanone 7,2′-dihydroxy-4′,5′-dimethoxyisoflavanone (**1**).

## 2. Results and Discussion

**Figure 2:**
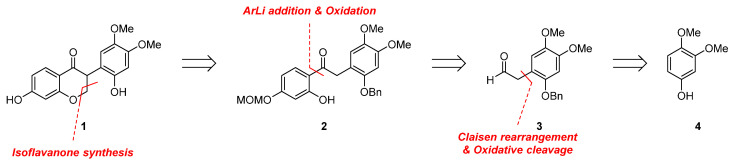
Retrosynthetic analysis of 7,2′-dihydroxy-4′,5′-dimethoxyisoflavanone (**1**).

Our approach for the synthesis of phytoestrogen 7,2′-dihydroxy-4′,5′-dimethoxyisoflavanone (**1**), as shown in Figure 2, includes the efficient construction of an isoflavanone framework and a gram-scale synthetic pathway. Isoflavanone **1** was obtained from 2-hydroxyketone **2** via the annulation of the deoxybenzoin skeleton and the sequential deprotection of the two masked phenols in the final stage. The deoxybenzoin unit of **2** can be constructed through a sequence of aryllithium additions to arylacetaldehyde **3** and the subsequent oxidation of the resulting alcohol. It was expected that **3** could be easily prepared from commercially available 3,4-dimethoxyphenol **4** via Claisen rearrangement and the subsequent oxidative cleavage of the terminal alkene to introduce a crucial acetaldehyde side chain.

**Scheme 1:**
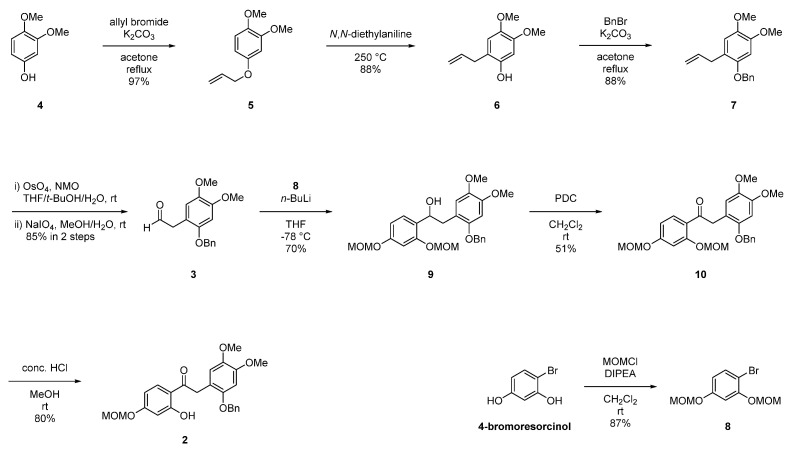
Preparation of key intermediate deoxybenzoin **2**.

The synthesis of **1** begins with the preparation of the key intermediate, deoxybenzoin **2**, as shown in Scheme 1. The allylation of commercially available 3,4-dimethoxyphenol **4** and subsequent Claisen rearrangement in *N*,*N*-diethylaniline [16] solely produced *o*-allyl-substituted phenol **6**, which was readily converted to benzyl ether **7**. The dihydroxylation of the terminal alkene of **7** and subsequent oxidative cleavage by NaIO_4_ readily afforded arylacetaldehyde **3**. Next, the lithiation of aryl bromide **8,** which was prepared from commercially available 4-bromoresorcinol, and a spontaneous nucleophilic addition to the acetaldehyde of **3** afforded benzyl alcohol **9**, which was converted to methoxymethyl (MOM)-protected deoxybenzoin **10** by pyridinium dichromate (PDC) oxidation. The selective MOM deprotection of the phenol adjacent to the ketone in deoxybenzoin **10** finally afforded 2-hydroxyketone **2**, which is a key intermediate for the construction of the isoflavanone framework of **1**.

**Scheme 2:**
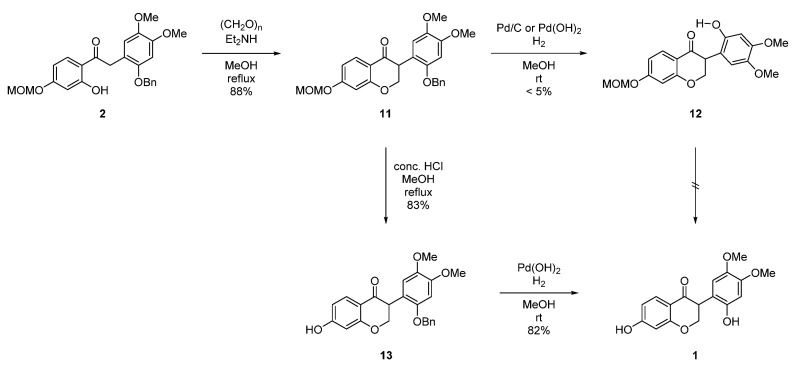
Completion of 7,2′-dihydroxy-4′,5′-dimethoxyisoflavanone (**1**) synthesis.

Using precursor **2**, the isoflavanone skeleton of **1** was constructed, as shown in Scheme 2. Previously, Gouda et al. reported an efficient and scalable approach to construct the isoflavanone framework [17]. Therefore, Gouda’s protocol was employed to obtain the fully functionalized isoflavanone **11** with paraformaldehyde and Et_2_NH in refluxing MeOH. As expected, isoflavanone **11** was obtained with a yield of 88% on a multi-gram scale. Finally, sequential deprotection reactions were performed for the two phenols in **11** with Bn and MOM protecting groups. However, initial attempts to remove the protecting groups of phenols were unsuccessful. In the presence of Pd/C or Pd(OH)_2_ (Pearlman’s catalyst), the hydrogenolysis of the benzyl group in **11** unexpectedly yielded phenol **12** with a very low yield (<5%). Furthermore, under acidic conditions for the MOM deprotection of **12**, phenol **12** was highly unstable and degradable, despite its structural simplicity. We assumed that the intrinsic structural instability of **12** was likely due to the presence of a free phenol moiety adjacent to the ketone in the 4-chromanone skeleton, leading to unexpected and inseparable messy mixtures, especially under acidic conditions. Therefore, the deprotection sequence for two masked phenols was changed, wherein the MOM ether was deprotected first, and the benzyl ether group was then cleaved under neutral conditions in the final stage via a hydrogenolysis reaction. Significantly, the careful deprotection of the MOM ether under acidic conditions afforded the desired phenol **13** without any degradation. Finally, the subsequent hydrogenolysis of the benzyl-protecting group successfully furnished the 7,2′-dihydroxy-4′,5′-dimethoxyisoflavanone (**1**) on a gram scale. The spectral data for synthetic **1** were identical to the reported data for the natural product in all aspects [13].

## 3. Conclusions

A concise and scalable synthesis of phytoestrogen 7,2′-dihydroxy-4′,5′-dimethoxyisoflavanone (**1**) was performed successfully. The key features of the synthesis include a deoxybenzoin intermediate obtained via a sequence of Claisen rearrangement and oxidative cleavage for the installation of the acetaldehyde side chain and the subsequent nucleophilic addition of functionalized aryllithium. Moreover, the scalable synthesis of the isoflavanone framework from the functionalized 2-hydroxyketone enabled the completion of isoflavanone **1** synthesis. Further *in vivo* studies on the hair growth effects and the elucidation of the therapeutic potential of phytoestrogen **1** as a promising treatment for hair loss diseases, such as alopecia, are currently in progress.

## 4. Materials and Methods

### 4.1. General Information

Unless noted otherwise, all starting materials and reagents were obtained from commercial suppliers and were used without further purification. All solvents used for the routine isolation of products and chromatography were reagent grade and glass-distilled. Reaction flasks were dried at 100 °C. Air- and moisture-sensitive reactions were performed under an argon atmosphere. Flash column chromatography was performed using silica gel 60 (230–400 mesh, Merck, Darmstadt, Germany) with the indicated solvents. Thin-layer chromatography was performed using 0.25 mm silica gel plates (Merck). High-resolution mass data were recorded by JMS-700 (JEOL, Tokyo, Japan), and methanol solvent was used to measure the MS-ESI spectra. Infrared (IR) spectra were measured on a 1600 FTIR spectrometer (Perkin-Elmer, Waltham, MA, USA). ^1^H and ^13^C NMR spectra were recorded on JEOL-500 (JEOL, Tokyo, Japan) as solutions in deuteriochloroform (CDCl_3_) and hexadeuterodimethyl sulfoxide (DMSO-d_6_). The melting point (m.p.) was measured using Electrothermal IA9100. Chemical shifts are expressed in parts per million (ppm, δ) downfield from tetramethylsilane and are referenced to the deuterated solvents (CHCl_3_ or HCD_2_SOCD_3_ for ^1^H NMR and CDCl_3_ or DMSO-d_6_ for ^13^C NMR). ^1^H NMR data are reported in the order of chemical shift, multiplicity (s, singlet; d, doublet; t, triplet; dd, doublet of doublets; dt, doublet of triplet; ddd, doublet of doublet of doublets; ddt, doublet of doublet of triplets; bs, broad singlet; m, multiplet and/or multiple resonance), number of protons, and coupling constant in hertz (Hz).

### 4.2. 4-(Allyloxy)-1,2-dimethoxybenzene (***5***)

To a solution of 3,4-dimethoxyphenol (10.0 g, 64.9 mmol) in acetone (120 mL) were added allyl bromide (8.43 mL, 97.5 mmol) and K_2_CO_3_ (22.5 g, 162.5 mmol) at ambient temperature. The reaction mixture was heated to reflux. After stirring for 12 h, the reaction mixture was cooled to ambient temperature. The resulting mixture was quenched with H_2_O (200 mL) and diluted with EtOAc (100 mL). The organic layer was separated, and the aqueous layer was extracted with EtOAc (2 × 50 mL). The combined organic layer was washed with brine, dried over MgSO_4_, and concentrated in vacuo. The residue was purified by flash column chromatography on silica gel (EtOAc/*n*-hexane = 1:10) to afford allyl ether **5** (12.2 g, 97%) as a colorless oil: ^1^H NMR (500 MHz, CDCl_3_) *δ* 6.76 (d, *J* = 9.2 Hz, 1H), 6.54 (d, *J* = 2.9 Hz, 1H), 6.39 (dd, *J* = 8.6, 2.9 Hz, 1H), 6.04 (ddt, *J* = 17.2, 10.3, 5.7 Hz, 1H), 5.40 (ddd, *J* = 17.2, 2.9, 1.7 Hz, 1H), 5.27 (ddd, *J* = 10.3, 2.9, 1.2 Hz, 1H), 4.48 (dt, *J* = 5.7, 1.4 Hz, 2H), 3.84 (s, 3H), 3.82 (s, 3H); ^13^C NMR (CDCl_3_, 125 MHz) *δ* 153.3, 149.9, 143.6, 133.6, 117.7, 111.7, 104.0, 101.2, 69.5, 56.5, 55.9; FT-IR (thin film, neat) *ν*_max_ 1597, 1510, 1425, 1228, 1197, 752 cm^−1^; HRMS (ESI+) calcd for C_11_H_15_O_3_ (M + H^+^) 195.1016, found 195.1013.

### 4.3. 2-Allyl-4,5-dimethoxyphenol (***6***)

Allyl ether **5** (12.2 g, 62.9 mmol) was dissolved in *N*,*N*-diethylaniline (250 mL) at ambient temperature. The reaction mixture was heated to 250 °C, stirred for 2 h, and cooled to ambient temperature. The solvent was removed by vacuum distillation. The crude residue was purified by flash column chromatography on silica gel (EtOAc/*n*-hexane = 1:4) to afford phenol **6** (10.8 g, 88%) as a colorless oil: ^1^H NMR (500 MHz, CDCl_3_) *δ* 6.60 (s, 1H), 6.44 (s, 1H), 5.97 (ddt, *J* = 17.8, 9.8, 3.5 Hz, 1H), 5.15-5.10 (m, 3H), 3.80 (s, 3H), 3.77 (s, 3H), 3.32 (d, *J* = 6.3 Hz, 2H); ^13^C NMR (CDCl_3_, 125 MHz) *δ* 148.5, 148.2, 143.0, 136.7, 116.3, 116.0, 114.0, 101.3, 56.7, 56.0, 34.8; FT-IR (thin film, neat) *ν*_max_ 3450, 1637, 1616, 1519, 1450, 1411, 1199, 1112, 997 cm^−1^; HRMS (ESI+) calcd for C_11_H_15_O_3_ (M + H^+^) 195.1016, found 195.1012.

### 4.4. 1-Allyl-2-(benzyloxy)-4,5-dimethoxybenzene (***7***)

To a solution of phenol **6** (10.8 g, 55.6 mmol) in acetone (110 mL) were added benzyl bromide (9.9 mL, 83.4 mmol) and K_2_CO_3_ (15.4 g, 111.2 mmol). The reaction mixture was heated to reflux. After stirring for 12 h, the reaction mixture was cooled to ambient temperature. The resulting mixture was quenched with H_2_O (30 mL) and diluted with EtOAc (50 mL). The organic layer was separated, and the aqueous layer was extracted with EtOAc (2 × 50 mL). The combined organic layer was washed with brine, dried over MgSO_4_, and concentrated in vacuo. The residue was purified by flash column chromatography on silica gel (EtOAc/*n*-hexane = 1:15) to afford benzyl ether **7** (13.9 g, 88%) as a colorless oil: ^1^H NMR (500 MHz, CDCl_3_) *δ* 7.43 (d, *J* = 7.5 Hz, 2H), 7.38 (t, *J* = 7.4 Hz, 2H), 7.31 (t, *J* = 7.2 Hz, 1H), 6.71 (s, 1H), 6.57 (s, 1H), 5.97 (ddt, *J* = 16.6, 10.3, 6.3 Hz, 1H), 5.07-5.03 (m, 4H), 3.83 (s, 3H), 3.83 (s, 3H), 3.38 (d, *J* = 6.3 Hz, 2H); ^13^C NMR (125 MHz, CDCl_3_) *δ* 150.4, 147.9, 143.5, 137.6, 137.4, 128.6, 127.9, 127.4, 120.8, 115.4, 113.8, 99.9, 71.7, 56.6, 56.3, 34.0; FT-IR (thin film, neat) *ν*_max_ 1608, 1514, 1448, 1222, 1193, 1118, 1026 cm^−1^; HRMS (ESI+) calcd for C_18_H_21_O_3_ (M + H^+^) 285.1485, found 285.1481.

### 4.5. 2-(2-(Benzyloxy)-4,5-dimethoxyphenyl)acetaldehyde (***3***)

To a solution of benzyl ether **7** (13.9 g, 49.0 mmol) in THF/*t*-BuOH/H_2_O (5:1:1, 70 mL) were added 4-methylmorpholine *N*-oxide (NMO) (6.89 g, 58.8 mmol) and OsO_4_ (0.1 M solution in toluene, 5 mL, 0.5 mmol) at ambient temperature. After stirring for 5 h, the resulting mixture was quenched with sat. Na_2_S_2_O_3_ solution (50 mL) and diluted with H_2_O (50 mL) and EtOAc (50 mL). The organic layer was separated, and the aqueous layer was extracted with EtOAc (3 × 50 mL). The combined organic layer was washed with brine, dried over MgSO_4_, and concentrated in vacuo. The residue was purified by flash column chromatography on silica gel (EtOAc only) to afford diol **S1** (13.3 g, 85%) as a white solid: m.p. 96–98 °C; ^1^H NMR (500 MHz, CDCl_3_) *δ* 7.41-7.32 (m, 5H), 6.71 (s, 1H), 6.57 (s, 1H), 5.03 (s, 2H), 3.88-3.85 (m, 1H), 3.82 (s, 6H), 3.55 (dd, *J* = 11.5, 3.4 Hz, 1H), 3.45 (dd, *J* = 11.5, 5.8 Hz, 1H), 2.81 (dd, *J* = 13.7, 5.7 Hz, 1H), 2.76 (dd, *J* = 13.7, 7.5 Hz, 1H), 2.26 (bs, 2H); ^13^C NMR (125 MHz, CDCl_3_) *δ* 150.7, 148.3, 143.6, 136.8, 128.8, 128.3, 127.6, 118.0, 115.0, 99.5, 72.6, 71.9, 65.8, 56.6, 56.3, 34.1; FT-IR (thin film, neat) *ν*_max_ 3425, 2935, 1610, 1514, 1382, 1219, 1193, 1078 cm^−1^; HRMS (ESI+) calcd for C_18_H_22_O_5_Na (M + Na^+^) 341.1359, found 341.1357.

To a solution of diol **S1** (13.3 g, 41.7 mmol) in MeOH/H_2_O (10:1, 55 mL) was added NaIO_4_ (10.7 g, 50.0 mmol) at ambient temperature. After stirring for 1 h, the reaction mixture was filtered through a pad of Celite and concentrated in vacuo. The crude residue was diluted with H_2_O (100 mL) and EtOAc (100 mL). The organic layer was separated, and the aqueous layer was extracted with EtOAc (3 × 50 mL). The combined organic layer was washed with brine, dried over MgSO_4_, and concentrated in vacuo. The residue was purified by flash column chromatography on silica gel (EtOAc/*n*-hexane = 1:1) to afford aldehyde **3** (11.9 g, 99%) as a colorless oil: ^1^H NMR (500 MHz, DMSO-*d*_6_) *δ* 9.54 (s, 1H), 7.38-7.27 (m, 5H), 6.82 (s, 1H), 6.79 (s, 1H), 5.04 (s, 2H), 3.73 (s, 3H), 3.65 (s, 3H), 3.55 (s, 2H); ^13^C NMR (125 MHz, DMSO-*d*_6_) *δ* 201.2, 151.1, 149.2, 143.4, 137.8, 128.9, 128.3, 128.0, 116.3, 113.4, 100.3, 70.8, 56.7, 56.4, 44.9; FT-IR (thin film, neat) *ν*_max_ 2827, 2723, 1606, 1512, 1398, 1220, 923 cm^−1^; HRMS (ESI+) calcd for C_17_H_19_O_4_ (M + H^+^) 287.1278, found 287.1277.

### 4.6. 1-Bromo-2,4-bis(methoxymethyl)benzene (***8***)

To a solution of 4-bromoresorcinol (7.4 g, 25.8 mmol) in CH_2_Cl_2_ (100 mL) were added DIPEA (14.4 mL, 50.0 mmol) and MOMCl (10.1 mL, 50.0 mmol) at 0 °C. After stirring for 3 h at ambient temperature, the resulting mixture was quenched with H_2_O (100 mL). The organic layer was separated, and the aqueous layer was extracted with CH_2_Cl_2_ (3 × 50 mL). The combined organic layer was washed with brine, dried over MgSO_4_, and concentrated in vacuo. The residue was purified by flash column chromatography on silica gel (EtOAc/*n*-hexane = 1:1) to afford aryl bromide **8** (11.9 g, 86%) as a colorless oil: ^1^H NMR (500 MHz, CDCl_3_) *δ* 7.40 (d, *J* = 8.6 Hz, 1H), 6.85 (d, *J* = 2.3 Hz, 1H), 6.61 (dd, *J* = 8.6, 2.9 Hz, 1H), 5.22 (s, 2H), 5.13 (s, 2H), 3.51 (s, 3H), 3.46 (s, 3H); ^13^C NMR (125 MHz, CDCl_3_) *δ* 157.7, 154.5, 133.3, 110.7, 105.5, 104.9, 95.2, 94.7, 56.5, 56.2; FT-IR (thin film, neat) *ν*_max_ 1587, 1481, 1274, 1082, 1037, 921 cm^−1^; HRMS (ESI+) calcd for C_11_H_14_O_4_Br (M + H^+^) 277.0070, found 277.0070.

### 4.7. 2-(2-(Benzyloxy)-4,5-dimethoxyphenyl)-1-(2,4-bis(methoxymethyl)phenyl)ethan-1-ol (***9***)

To a solution of 1-bromo-2,4-bis(methoxymethyl)benzene **8** (8.2 g, 29.5 mmol) in dry THF (100 mL) was added *n*-BuLi (2.5 M in *n*-hexane, 13.2 mL, 32.4 mmol) at −78 °C. After stirring for 30 min at the same temperature, aldehyde **3** (7.4 g, 25.8 mmol) in THF (30 mL) was added to this mixture and stirred for an additional 2 h. The resulting mixture was quenched with sat. NH_4_Cl solution (100 mL) and diluted with EtOAc (50 mL). The organic layer was separated, and the aqueous layer was extracted with EtOAc (3 × 50 mL). The combined organic layer was washed with brine, dried over MgSO_4_, and concentrated in vacuo. The residue was purified by flash column chromatography on silica gel (EtOAc/*n*-hexane = 1:3) to afford alcohol **9** (8.75 g, 70%) as a colorless oil: ^1^H NMR (500 MHz, CDCl_3_) *δ* 7.43 (d, *J* = 6.9 Hz, 2H), 7.38 (t, *J* = 7.5 Hz, 2H), 7.32 (m, 1H), 7.17 (d, *J* = 8.0 Hz, 1H), 6.74 (d, *J* = 2.3 Hz, 1H), 6.63 (dd, *J* = 8.6, 2.3 Hz, 1H), 6.58 (s, 1H), 6.55 (s, 1H), 5.14-5.11 (m, 3H), 5.02 (s, 2H), 5.00 (d, *J* = 2.3 Hz, 2H), 3.81 (s, 3H), 3.75 (s, 3H), 3.45 (s, 3H), 3.38 (s, 3H), 3.10 (dd, *J* = 10.7, 4.6 Hz, 1H), 3.00 (dd, *J* = 13.8, 8.0 Hz, 1H); ^13^C NMR (125 MHz, CDCl_3_) *δ* 157.4, 154.9, 151.0, 148.1, 143.2, 137.3, 128.7, 128.1, 127.7, 126.7, 119.1, 115.2, 108.8, 103.2, 99.4, 94.6, 94.4, 71.6, 70.3, 56.5, 56.2, 56.1, 56.1, 38.5; FT-IR (thin film, neat) *ν*_max_ 3523, 2953, 1610, 1519, 1219, 1153, 1008 cm^−1^; HRMS (ESI+) calcd for C_27_H_32_O_8_Na (M + Na^+^) 507.1989, found 507.1990.

### 4.8. 2-(2-(Benzyloxy)-4,5-dimethoxyphenyl)-1-(2,4-bis(methoxymethyl)phenyl)ethan-1-one (***10***)

To a solution of alcohol **9** (7.4 g, 14.9 mmol) in dry CH_2_Cl_2_ (150 mL) was added PDC (13.2 g, 32.4 mmol) at ambient temperature. After stirring for 12 h, the reaction mixture was filtered through a pad of Celite and concentrated in vacuo. The residue was purified by flash column chromatography on silica gel (1% CH_2_Cl_2_ in EtOAc/*n*-hexane = 1:4) to afford ketone **10** (3.68 g, 51%) as a colorless oil: ^1^H NMR (500 MHz, CDCl_3_) *δ* 7.69 (d, *J* = 9.2 Hz, 1H), 7.28-7.25 (m, 5H), 6.79 (d, *J* = 2.3 Hz, 1H), 6.73 (s, 1H), 6.66 (dd, *J* = 8.6, 2.3 Hz, 1H), 6.56 (s, 1H), 5.18 (s, 2H), 5.17 (s, 2H), 4.97 (s, 2H), 4.24 (s, 2H), 3.82 (s, 3H), 3.81 (s, 3H) 3.47 (s, 3H), 3.41 (s, 3H); ^13^C NMR (125 MHz, CDCl_3_) *δ* 198.6, 161.5, 158.0, 150.8, 148.4, 143.2, 137.4, 132.4, 128.5, 127.8, 127.4, 123.0, 116.7, 114.9, 108.9, 102.9, 99.5, 94.6, 94.3, 71.5, 56.5, 56.5, 56.4, 56.2, 44.5; FT-IR (thin film, neat) *ν*_max_ 1673, 1600, 1516, 1247, 1195, 1153, 1002 cm^−1^; HRMS (FAB+) calcd for C_27_H_30_O_8_ (M + H^+^) 482.1941, found 482.1939.

### 4.9. 2-(2-(Benzyloxy)-4,5-dimethoxyphenyl)-1-(2-hydroxy-4-(methoxymethyl)phenyl)ethan-1-one (***2***)

To a solution of ketone **10** (3.68 g, 7.62 mmol) in MeOH (100 mL) was added conc. HCl (0.1 mL) at ambient temperature. After stirring for 3 h at the same temperature, solvents were partially evaporated to 20 mL under reduced pressure. After the residue was triturated with EtOAc/*n*-hexane (1:10) for 30 min, the resulting precipitate was filtered and dried to afford 2-hydroxyketone **2** (2.67 g, 80%) as a white solid: m.p. 143-144 °C; ^1^H NMR (500 MHz, DMSO-*d_6_*) *δ* 12.39 (s, 1H), 7.95 (d, *J* = 9.8 Hz, 1H), 7.25-7.21 (m, 5H), 6.83 (s, 1H), 6.75 (s, 1H), 6.50-6.48 (m, 2H), 5.23 (s, 2H), 5.00 (s, 2H), 4.19 (s, 2H), 3.72 (s, 3H), 3.64 (s, 3H) 3.35 (s, 3H); ^13^C NMR (125 MHz, CDCl_3_) *δ* 203.1, 165.2, 163.5, 150.3, 148.9, 143.5, 137.0, 132.4, 128.7, 128.1, 127.6, 115.0, 114.4, 114.2, 108.1, 103.8, 99.2, 94.0, 71.7, 56.6, 56.5, 56.2, 38.8; FT-IR (thin film, neat) *ν*_max_ 2904, 2835, 1627, 1521, 1392, 1220, 1083, 989, 923 cm^−1^; HRMS (FAB+) calcd for C_25_H_26_O_7_ (M^+^) 438.1679, found 438.1683.

### 4.10. 3-(2-(Benzyloxy)-4,5-dimethoxyphenyl)-7-(methoxymethyl)chroman-4-one (***11***)

To a solution of 2-hydroxyketone **2** (2.73 g, 6.23 mmol) in MeOH (100 mL) were added paraformaldehyde (561 mg, 18.8 mmol) and Et_2_NH (1.93 mL, 18.8 mmol) at ambient temperature. The reaction mixture was heated to reflux. After stirring for 2 h at the same temperature, the resulting mixture was cooled to ambient temperature and concentrated in vacuo. The crude residue was diluted with H_2_O (50 mL) and EtOAc (50 mL). The organic layer was separated, and the aqueous layer was extracted with EtOAc (3 × 20 mL). The combined organic layer was washed with brine, dried over MgSO_4_, and concentrated in vacuo. The residue was purified by flash column chromatography on silica gel (EtOAc/*n*-hexane = 1:3) to afford isoflavanone **11** (2.46 g, 88%) as a colorless oil: ^1^H NMR (500 MHz, CDCl_3_) *δ* 7.89 (d, *J* = 8.6 Hz, 1H), 7.33-7.24 (m, 5H), 6.68–6.66 (m, 2H), 6.59 (s, 1H), 6.58 (d, *J* = 2.3 Hz, 1H), 5.19 (s, 2H), 5.00 (s, 2H), 4.58 (dd, *J* = 12.0, 10.9 Hz, 1H), 4.44 (dd, *J* = 10.9, 5.8 Hz, 1H), 4.26 (dd, *J* = 12.0, 5.2 Hz, 1H), 3.82 (s, 3H), 3.79 (s, 3H), 3.47 (s, 3H); ^13^C NMR (125 MHz, CDCl_3_) *δ* 191.6, 163.6, 163.2, 151.0, 149.3, 143.5, 137.0, 129.5, 128.6, 128.0, 127.5, 116.4, 115.4, 114.2, 110.9, 103.4, 99.6, 94.1, 71.7, 71.2, 56.6, 56.4, 56.2, 48.2; FT-IR (thin film, neat) *ν*_max_ 1735, 1683, 1608, 1512, 1377, 1238, 1153, 1022 cm^−1^; HRMS (FAB+) calcd for C_26_H_27_O_7_ (M + H^+^) 451.1757, found 451.1754.

### 4.11. 3-(2-(Benzyloxy)-4,5-dimethoxyphenyl)-7-hydroxychroman-4-one (***13***)

To a solution of isoflavanone **11** (2.40 g, 5.33 mmol) in MeOH (100 mL) was added conc. HCl (0.1 mL). The reaction mixture was heated to reflux. After stirring for 4 h at the same temperature, the resulting mixture was cooled to ambient temperature, and MeOH was evaporated to 20 mL under reduced pressure. After the residue was triturated with EtOAc/*n*-hexane (1:3) for 30 min, the resulting precipitate was filtered and dried to afford phenol **13** (1.80 g, 83%) as a white solid: m.p. 193-194 °C; ^1^H NMR (500 MHz, DMSO-d_6_) *δ* 10.51 (s, 1H), 7.64 (d, *J* = 8.6 Hz, 1H), 7.30 (d, *J* = 6.3 Hz, 2H), 7.24-7.20 (m, 3H), 6.79 (s, 1H), 6.78 (s, 1H), 6.47 (dd, *J* = 8.6, 2.3 Hz, 1H), 6.28 (d, *J* = 2.3 Hz, 1H), 5.00 (q, *J* = 11.7 Hz, 2H), 4.51 (t, *J* = 11.5 Hz, 1H), 4.33 (dd, *J* = 10.9, 5.7 Hz, 1H), 4.17 (dd, *J* = 12.6, 5.7 Hz, 1H), 3.73 (s, 3H), 3.62 (s, 3H); ^13^C NMR (125 MHz, DMSO-d_6_) *δ* 190.8, 164.8, 163.7, 151.1, 149.4, 143.4, 137.6, 129.6, 128.8, 128.2, 128.0, 116.0, 114.6, 111.1, 102.9, 100.7, 71.1, 70.6, 56.9, 56.4, 48.1; FT-IR (thin film, neat) *ν*_max_ 3336, 1604, 1517, 1450, 1220, 1022 cm^−1^; HRMS (FAB+) calcd for C_24_H_22_O_6_ (M^+^) 406.1416, found 406.1421.

### 4.12. 7,2′-Dihydroxy-4′,5′-dimethoxyisoflavanone (***1***)

To a solution of phenol **13** (1.75 g, 4.31 mmol) in MeOH (100 mL) was added Pd(OH)_2_ (100 mg) at ambient temperature. After stirring for 2 h under H_2_ gas (balloon), the resulting mixture was filtered through a pad of Celite and concentrated in vacuo. The residue was purified by flash column chromatography on silica gel (CH_2_Cl_2_/MeOH = 10:1) to afford 7,2′-dihydroxy-4′,5′-dimethoxyisoflavanone **1** (1.12 g, 82%) as a white solid: m.p. 181-182 °C; ^1^H NMR (500 MHz, DMSO-*d*_6_) *δ* 10.62 (bs, 1H), 9.19 (bs, 1H), 7.65 (d, *J* = 8.6 Hz, 1H), 6.64 (s, 1H), 6.50 (dd, *J* = 8.9, 2.3 Hz, 1H), 6.45 (s, 1H), 6.32 (d, *J* = 2.3 Hz, 1H), 4.57 (t, *J* = 11.5 Hz, 1H), 4.37 (dd, *J* = 10.9, 5.2 Hz, 1H), 4.10 (dd, *J* = 12.1, 5.2 Hz, 1H), 3.67 (s, 3H), 3.58 (s, 3H); ^13^C NMR (125 MHz, DMSO-*d*_6_) *δ* 190.5, 164.4, 163.3, 149.5, 148.8, 141.5, 129.0, 115.4, 114.1, 112.7, 110.6, 102.4, 100.9, 70.1, 56.5, 55.4, 47.1; FT-IR (thin film, neat) *ν*_max_ 3300, 1598, 1514, 1459, 1267, 1199, 1107, 734 cm^−1^; HRMS (FAB+) calcd for C_17_H_16_O_6_ (M^+^) 316.0947, found 316.0947.

## Data Availability

The data are available in the manuscript and Supplementary Materials.

## Supplementary Materials

The following are available online at https://www.mdpi.com/article/10.3390/molecules27196660/s1. File S1: Comparison of NMR spectra of **1** and copies of NMR spectra (^1^H and ^13^C). Refs. [13,15,18] are cited in the Supplementary Materials.

## Sample Availability

Samples of the compounds are not available from the authors.
