# Supplementary material for: Synthesis of 7,2′-Dihydroxy-4′,5′-Dimethoxyisoflavanone, a Phytoestrogen with Derma Papilla Cell Proliferative Activity
Source: Molecules. 2022 Oct 7;27(19):6660. doi: 10.3390/molecules27196660 (PMC9572366; doi:10.3390/molecules27196660)
Supplement: Supplementary file 1 [file molecules-27-06660-s001.zip › molecules-1959028-supplementary.pdf]

Supplementary Information (File S1)

Synthesis of 7,2′-dihydroxy-4′,5′-dimethoxyisoflavanone, a phytoestrogen with derma papilla cell proliferative activity

Taewoo Kim [1,†], Hyun Su Kim [1,†], Jaebong Jang[2,†], Dong-Jun Kim [1], Jongkook Lee[3], Dongjoo Lee [4,*], and Seok-Ho Kim [3,*]

[1] College of Pharmacy and Institute of Pharmaceutical Sciences, CHA University, 120 Haeryong-ro, Pocheon-si, Gyeonggi-do, 11160, Republic of Korea

[2] College of Pharmacy, Korea University, Sejong 30019, Republic of Korea

[3] College of Pharmacy, Kangwon National University, Chuncheon, Gangwon-do 24341, Republic of Korea

[4] College of Pharmacy and Research Institute of Pharmaceutical Science and Technology (RIPST), Ajou University, 206 Worldcup-ro, Yeongtong-gu, Suwon 16499, Korea

* Correspondence: ksh3410@cha.ac.kr (S.-H.K.); dongjoo@ajou.ac.kr (D.L.)

[†] These authors contributed equally to this work.

List of Contents

I. Comparison of NMR spectra of **1**……………………..…..................S03

II. ^{1}H and ^{13}C NMR spectra………………………………….…..S04-S15

III. Comparison of Synthetic methods of **1**………………....…………….S16

IV. References……………………..…………….……………….…….……S17

I. Comparison of NMR spectra of 1

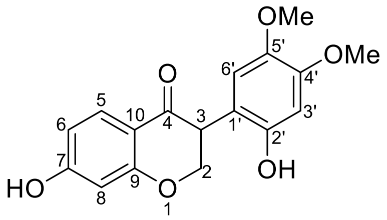

1

7,2′-dihydroxy-4′,5′-dimethoxyisoflavanone (**1**)

Position	^{1}H NMR (DMSO-d$_6$) of **1**		^{13}C NMR (DMSO-d$_6$) of **1**		
	Natural 7,2′-dihydroxy-4′,5′-dimethoxyisoflavanone[1]	Synthetic 7,2′-dihydroxy-4′,5′-dimethoxyisoflavanone	Natural 7,2′-dihydroxy-4′,5′-dimethoxyisoflavanone[1]	Synthetic 7,2′-dihydroxy-4′,5′-dimethoxyisoflavanone	
	δ_H (*J*, Hz) at 400 MHz	δ_H (*J*, Hz) at 500 MHz	δ_C at 100 MHz	δ_C at 125 MHz	Δ δ
2	ax; 4.55, dd (10.9, 12.0)<br>eq; 4.36, dd (10.9, 5.5)	ax; 4.57, t (11.5)<br>eq; 4.37, dd (10.9, 5.2)	70.1	70.1	0
3	4.10, dd (5.5, 12.0)	4.10, dd (5.2, 12.1)	47.0	47.1	0.1
4			190.5	190.5	0
5	7.65, d (8.6)	7.65, d (8.6)	128.9	129.0	0.1
6	6.49, dd (2.3, 8.6)	6.50, dd, (2.3, 8.9)	110.5	110.6	0.1
7			164.2	164.4	0.2
8	6.33, d (2.3)	6.32, d (2.3)	102.3	102.4	0.1
9			163.2	163.3	0.1
10			114.1	114.1	0
7-OH	10.50, s	10.62, bs			
1'			112.7	112.7	0
2'			149.5	149.5	0
3'	6.45, s	6.45, s	100.9	100.9	0
4'			148.8	148.8	0
5'			141.5	141.5	0
6'	6.65, s	6.64, s	115.4	115.4	0
2'-OH	9.13, s	9.19, bs			
4'-OMe	3.68, s	3.67, s	55.4	55.4	0
5'-OMe	3.59, s	3.58, s	56.5	56.5	0

Table S1. Comparison of the ^{1}H and ^{13}C NMR spectra of the reported 7,2′-dihydroxy-4′,5′-dimethoxyisoflavanone (**1**)[1] with that of synthetic **1**.

II. ^{1}H and ^{13}C NMR spectra

4-(Allyloxy)-1,2-dimethoxybenzene (**5**)

^{1}H NMR ($CDCl_3$, 500 MHz)

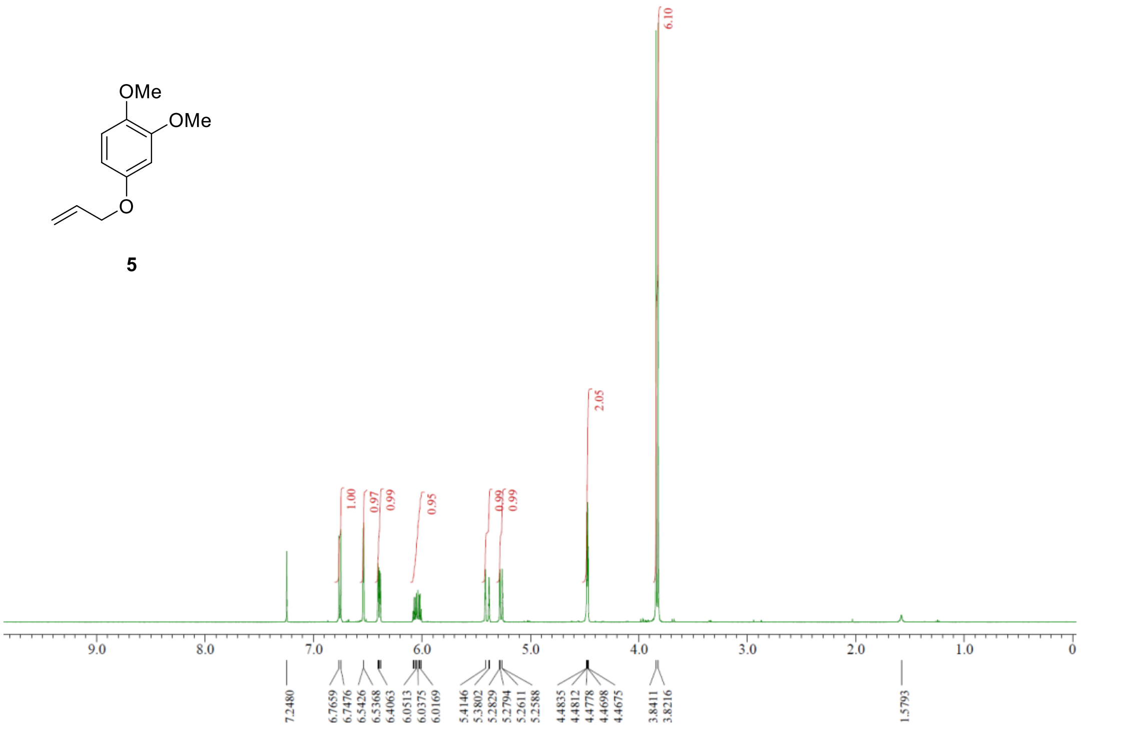

^{13}C NMR ($CDCl_3$, 125 MHz)

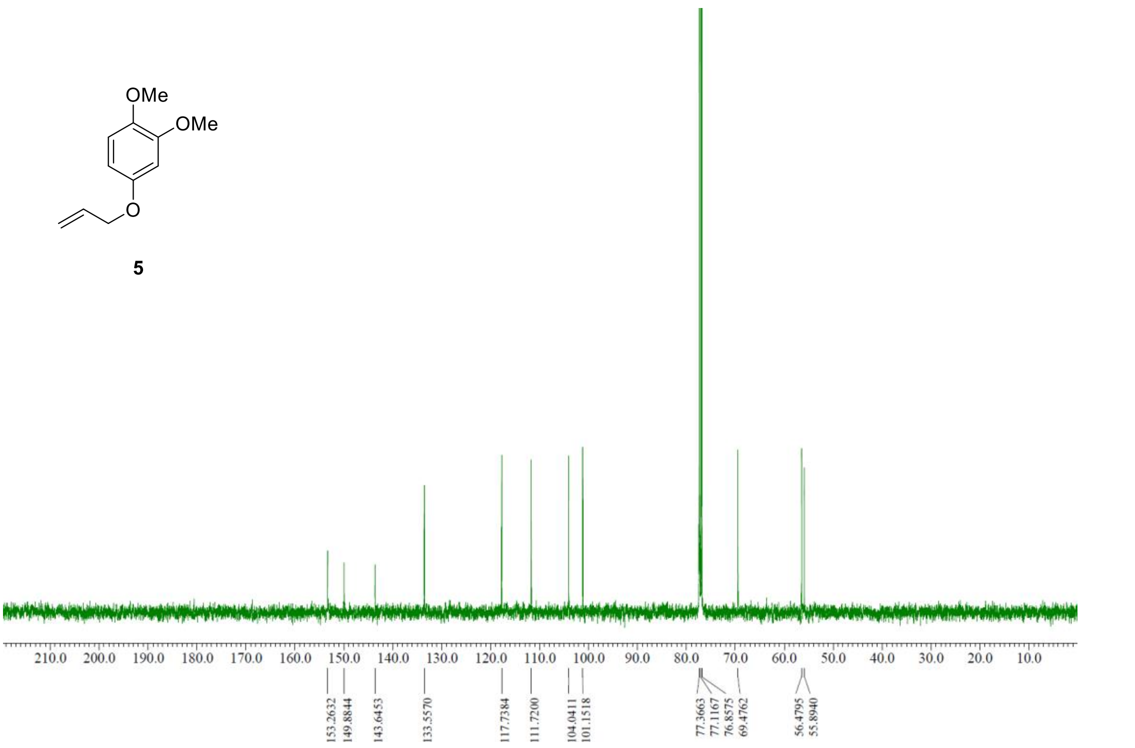

2-Allyl-4,5-dimethoxyphenol (**6**)

^{1}H NMR ($CDCl_3$, 500 MHz)

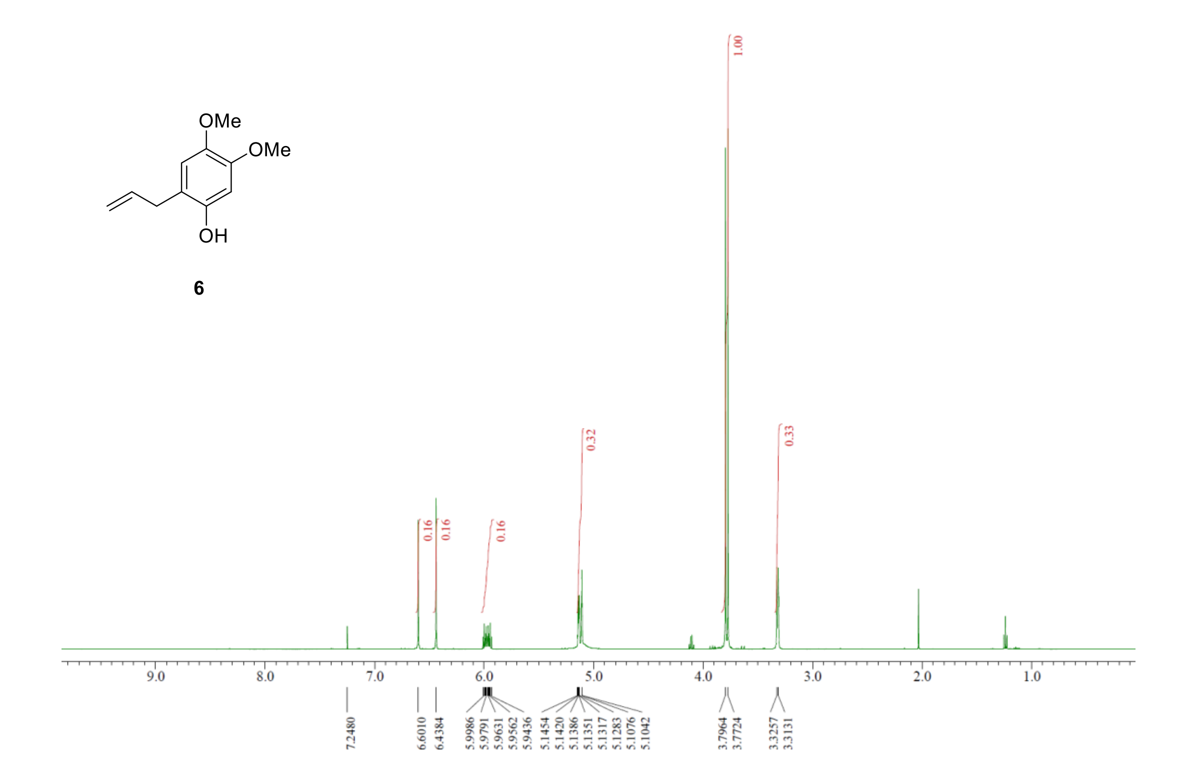

^{13}C NMR ($CDCl_3$, 125 MHz)

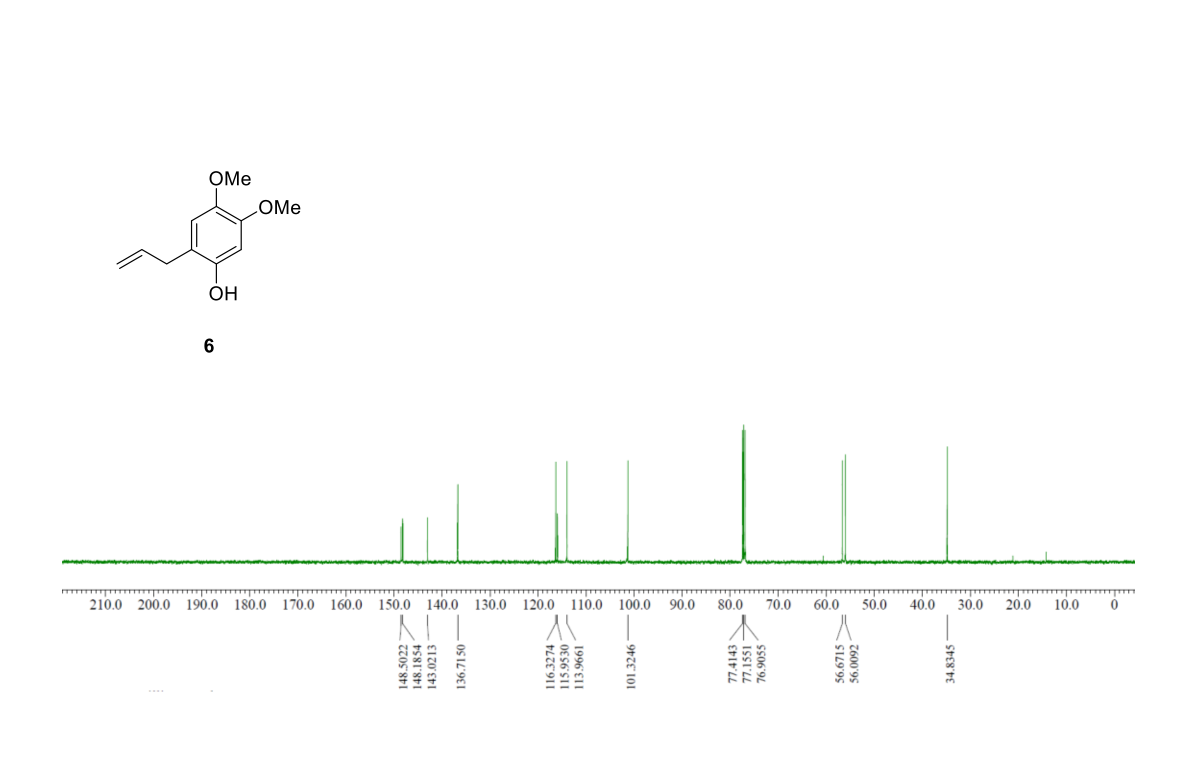

1-Allyl-2-(benzyloxy)-4,5-dimethoxybenzene (**7**)

^{1}H NMR ($CDCl_3$, 500 MHz)

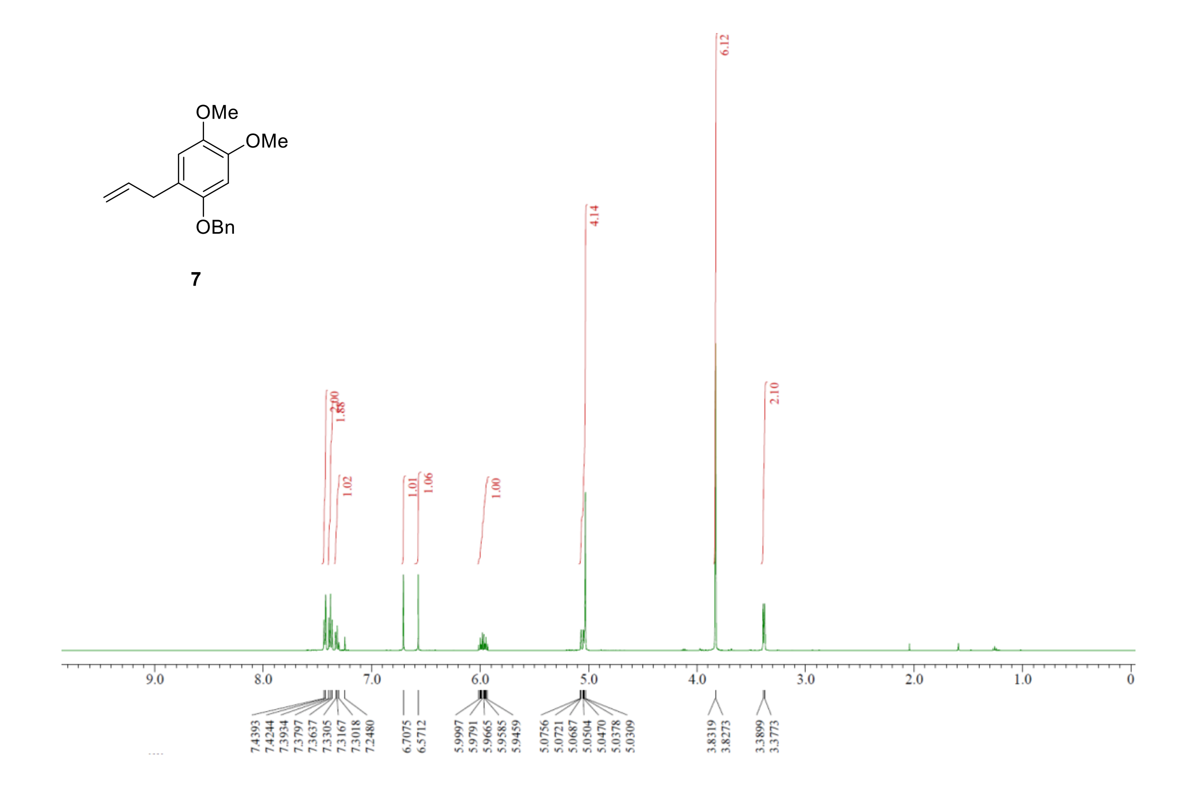

^{13}C NMR ($CDCl_3$, 125 MHz)

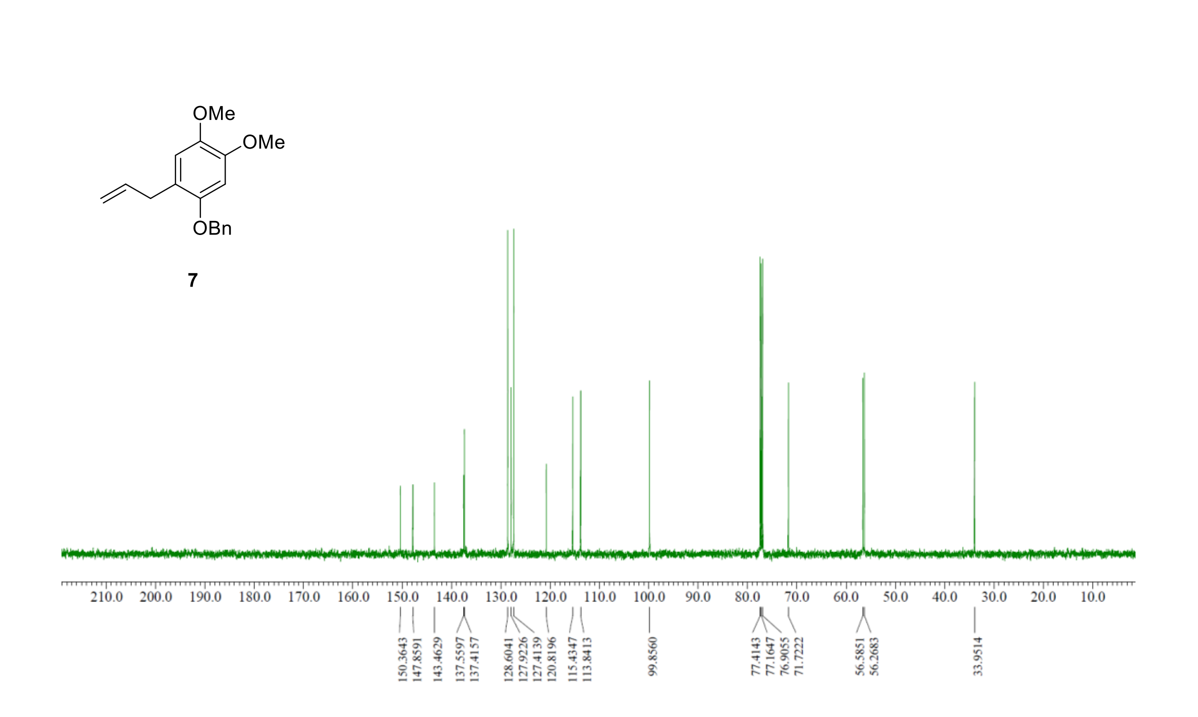

3-(2-(benzyloxy)-4,5-dimethoxyphenyl)propane-1,2-diol (**S1**)

^{1}H NMR ($CDCl_3$, 500 MHz)

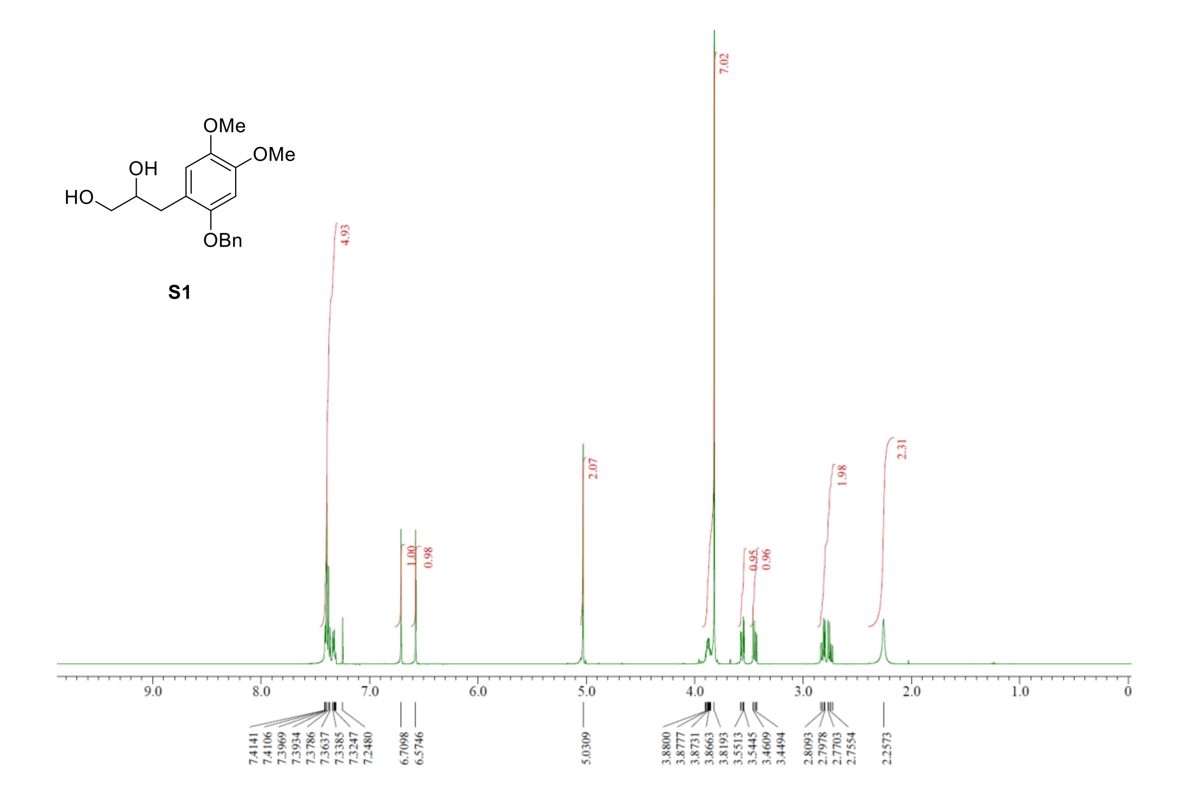

^{13}C NMR ($CDCl_3$, 125 MHz)

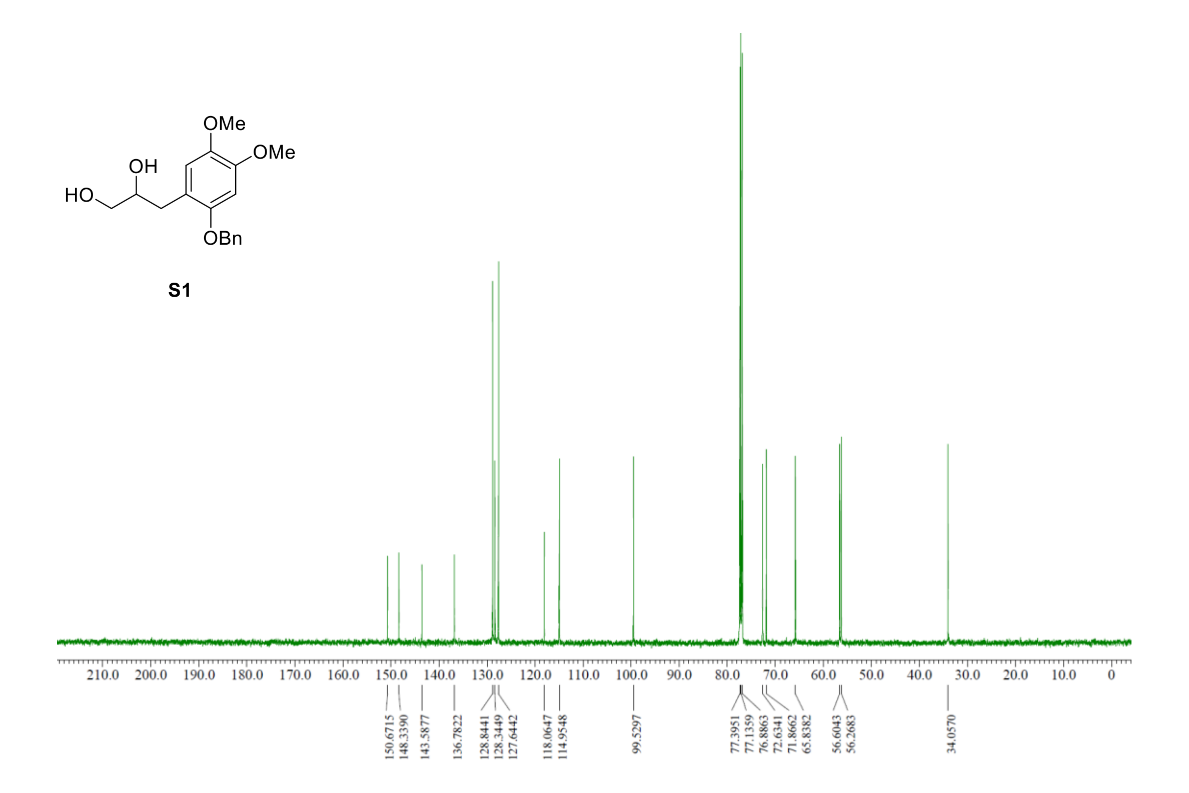

2-(2-(Benzyloxy)-4,5-dimethoxyphenyl)acetaldehyde (**3**)

^{1}H NMR (DMSO-d$_6$, 500 MHz)

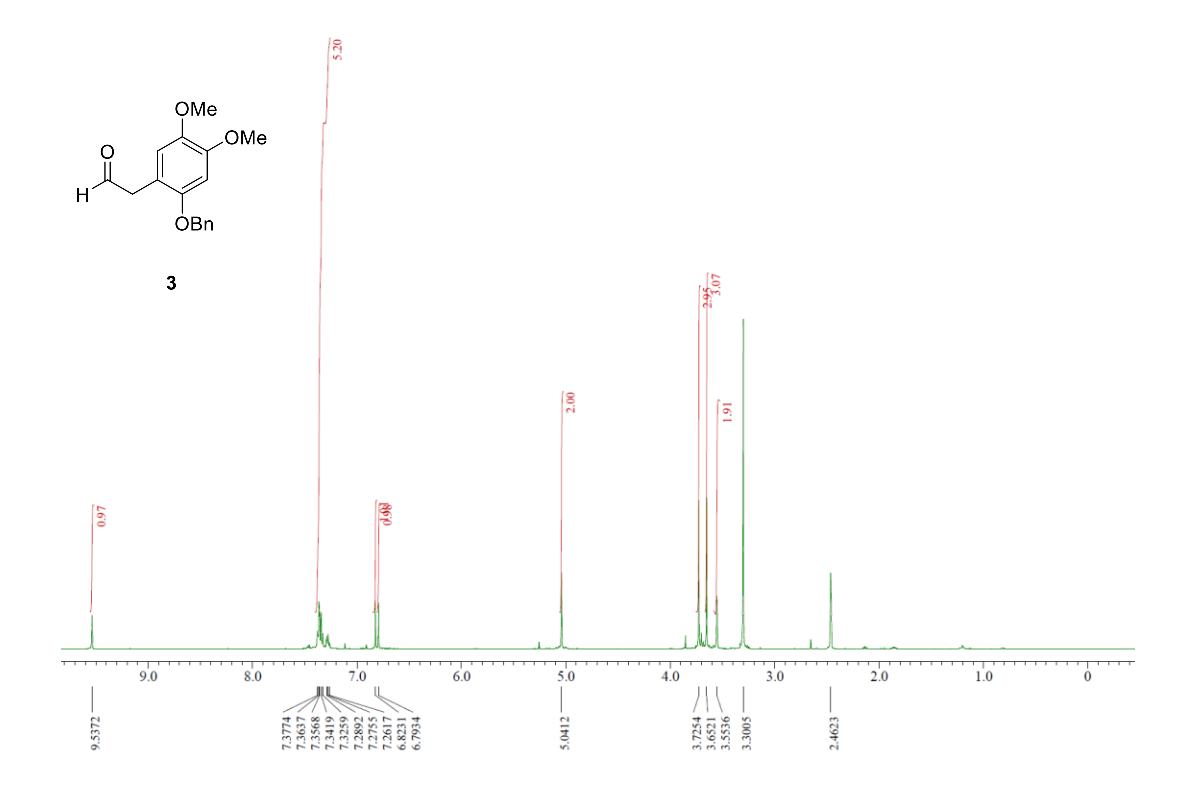

^{13}C NMR (DMSO-d$_6$, 125 MHz)

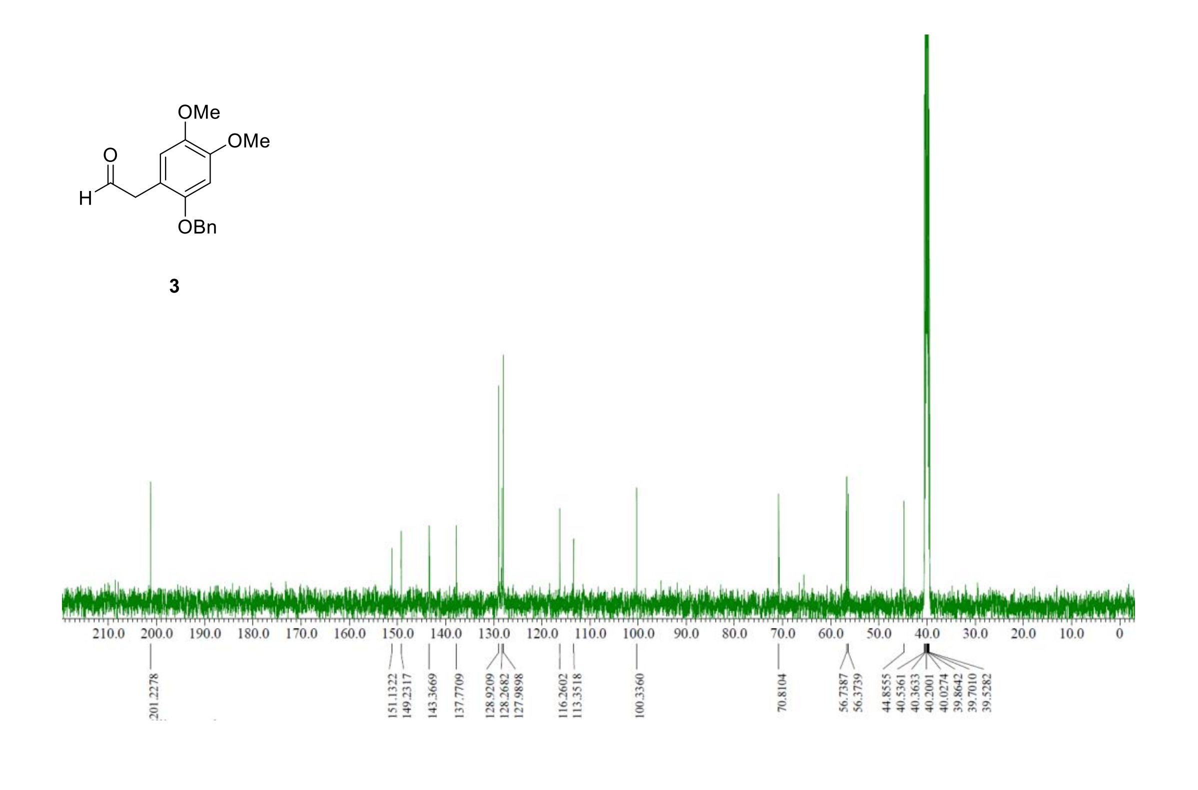

1-Bromo-2,4-bis(methoxymethoxy)benzene (**8**)

^{1}H NMR ($CDCl_3$, 500 MHz)

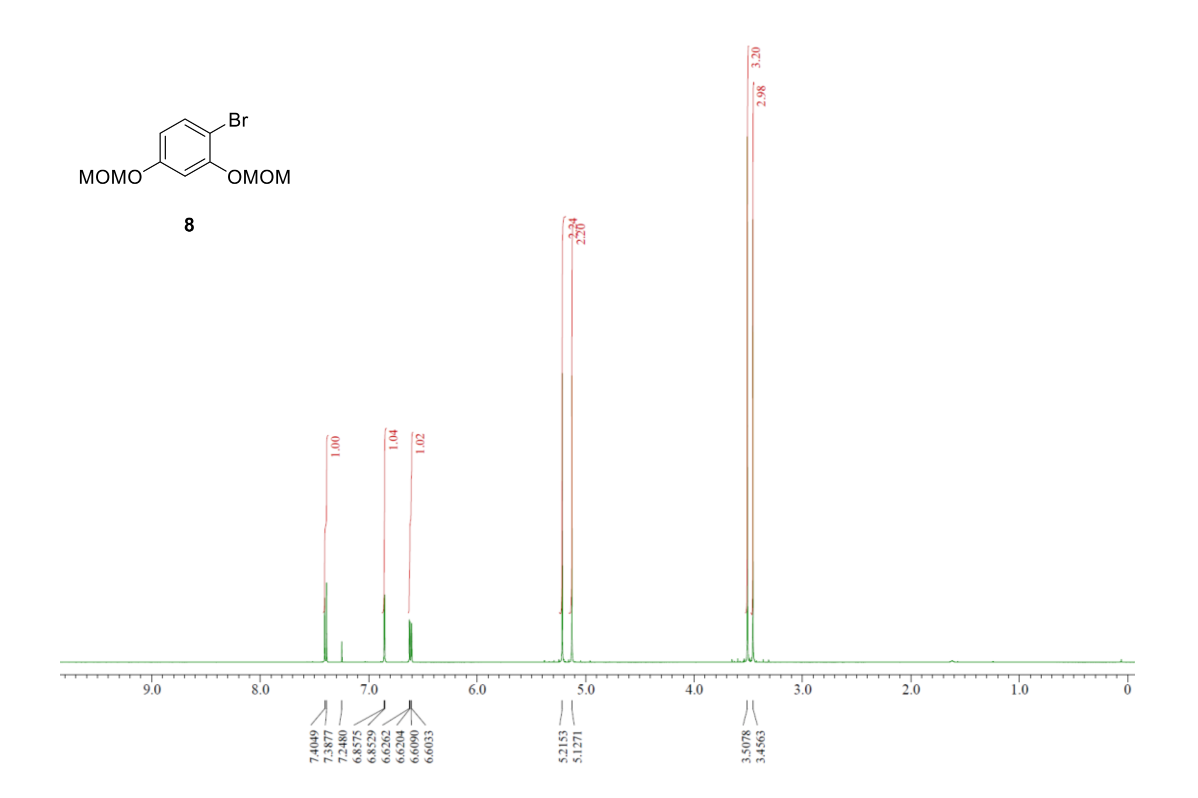

^{13}C NMR ($CDCl_3$, 125 MHz)

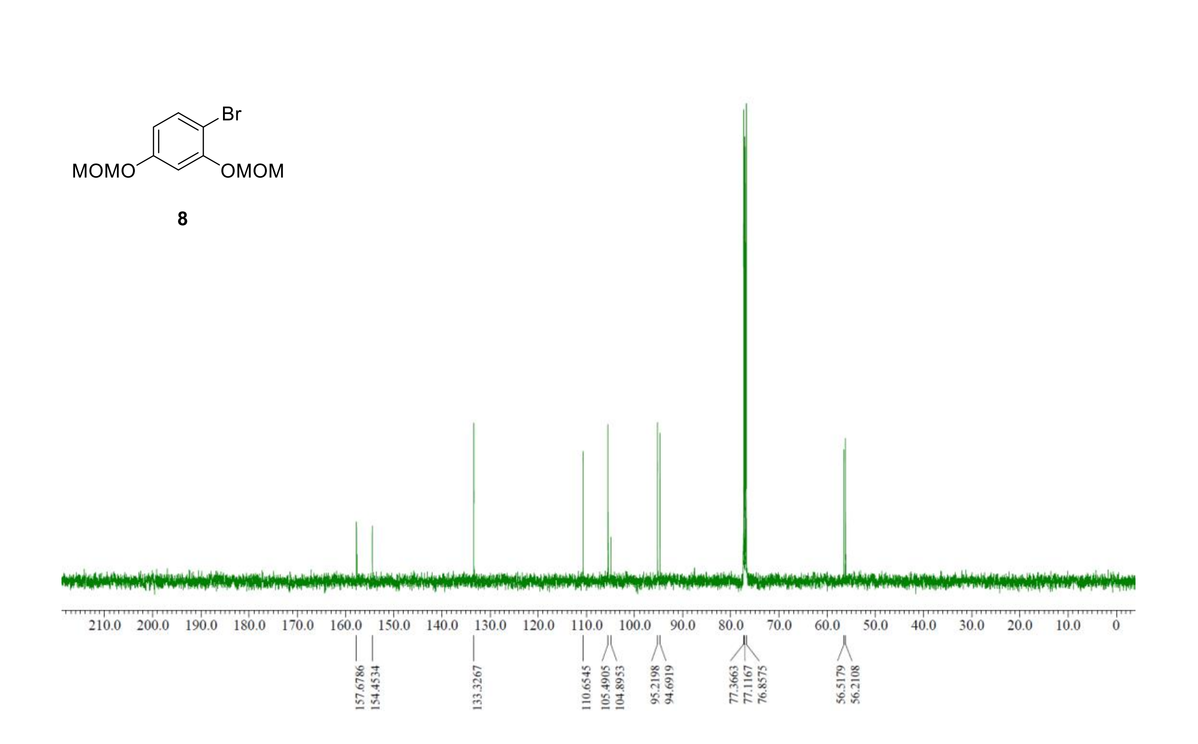

2-(2-(Benzyloxy)-4,5-dimethoxyphenyl)-1-(2,4-bis(methoxymethoxy)phenyl)ethan-1-ol (**9**)

^{1}H NMR ($CDCl_3$, 500 MHz)

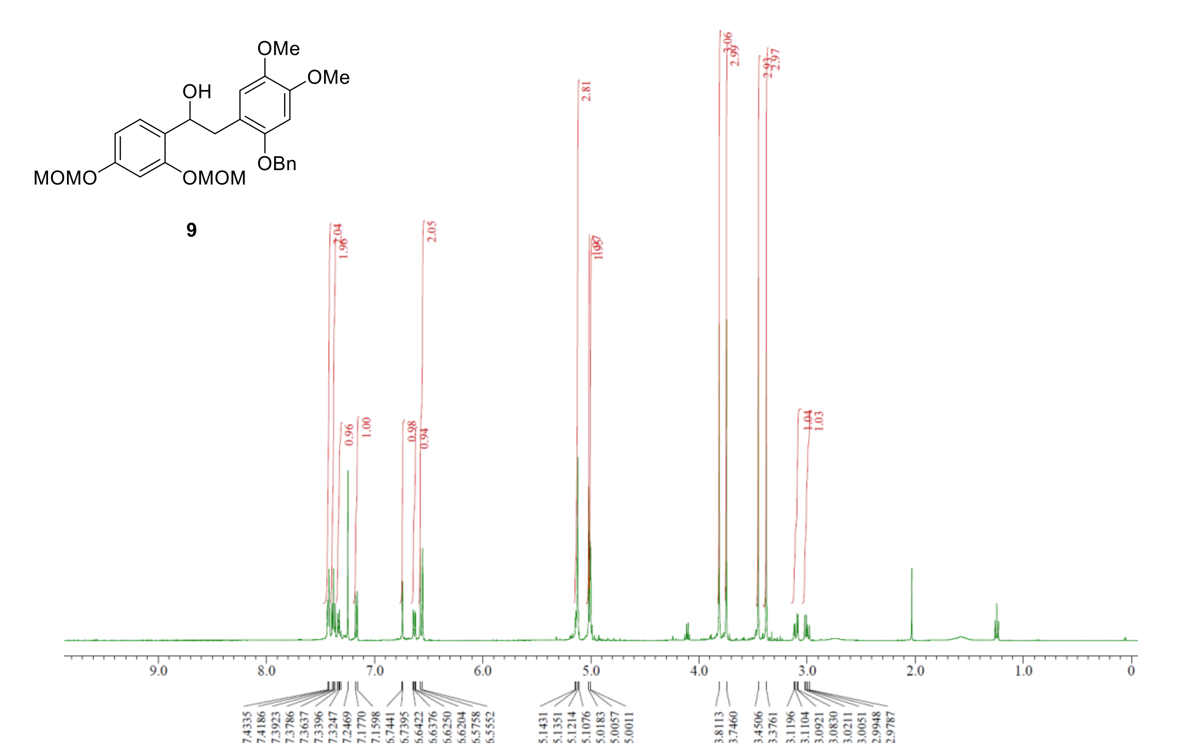

^{13}C NMR ($CDCl_3$, 125 MHz)

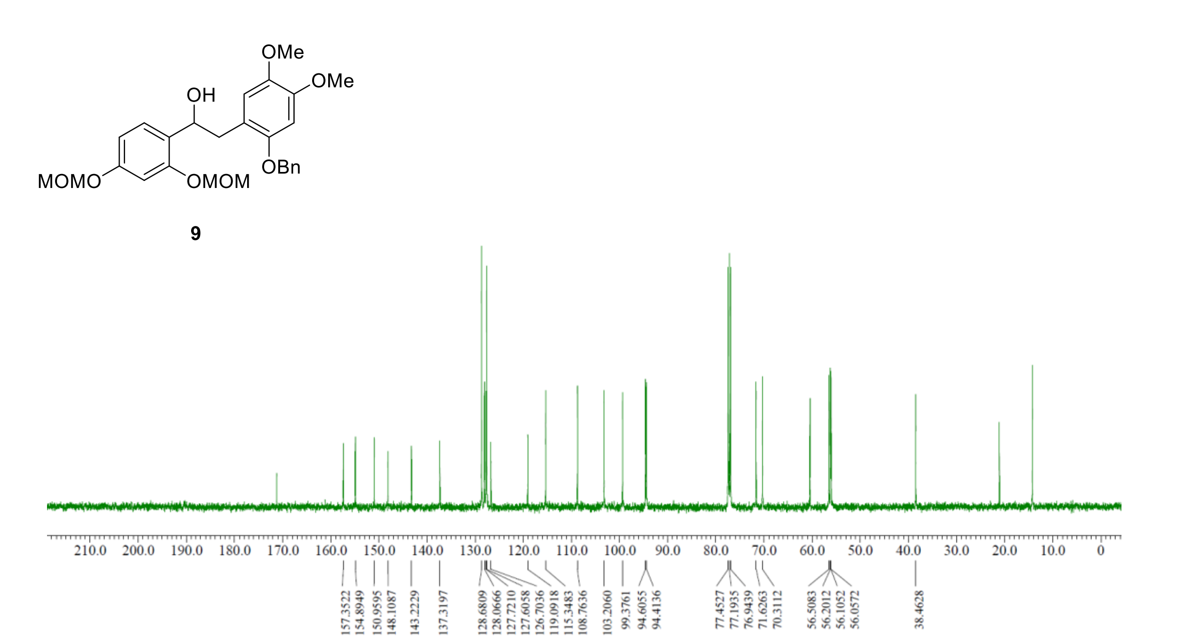

2-(2-(Benzyloxy)-4,5-dimethoxyphenyl)-1-(2,4-bis(methoxymethoxy)phenyl)ethan-1-one (**10**)

^{1}H NMR ($CDCl_3$, 500 MHz)

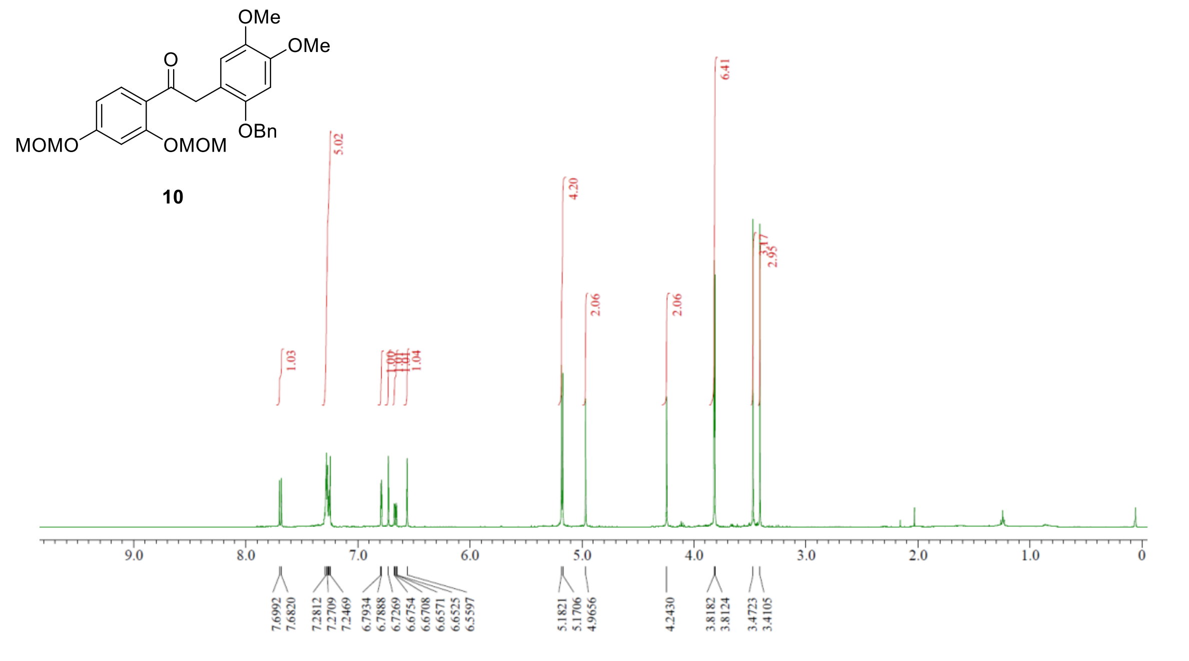

^{13}C NMR ($CDCl_3$, 125 MHz)

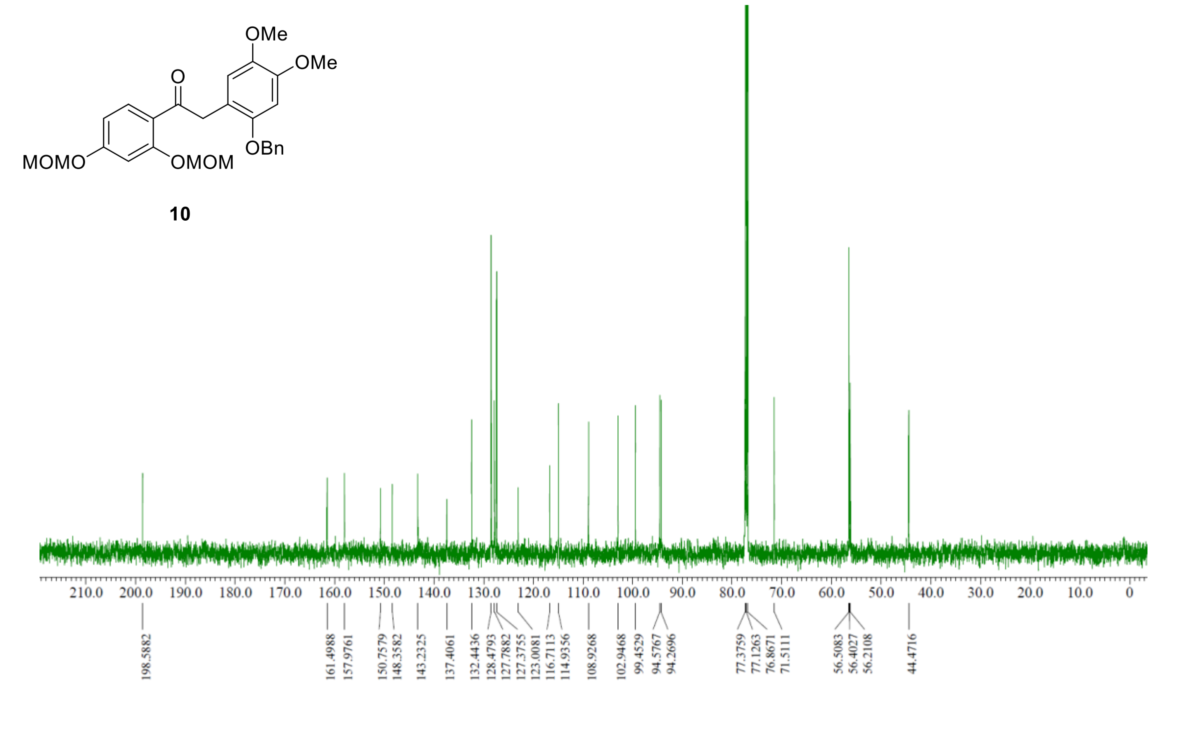

2-(2-(Benzyloxy)-4,5-dimethoxyphenyl)-1-(2-hydroxy-4-(methoxymethoxy)phenyl)ethan-1-one (**2**)

^{1}H NMR (DMSO-d$_6$, 500 MHz)

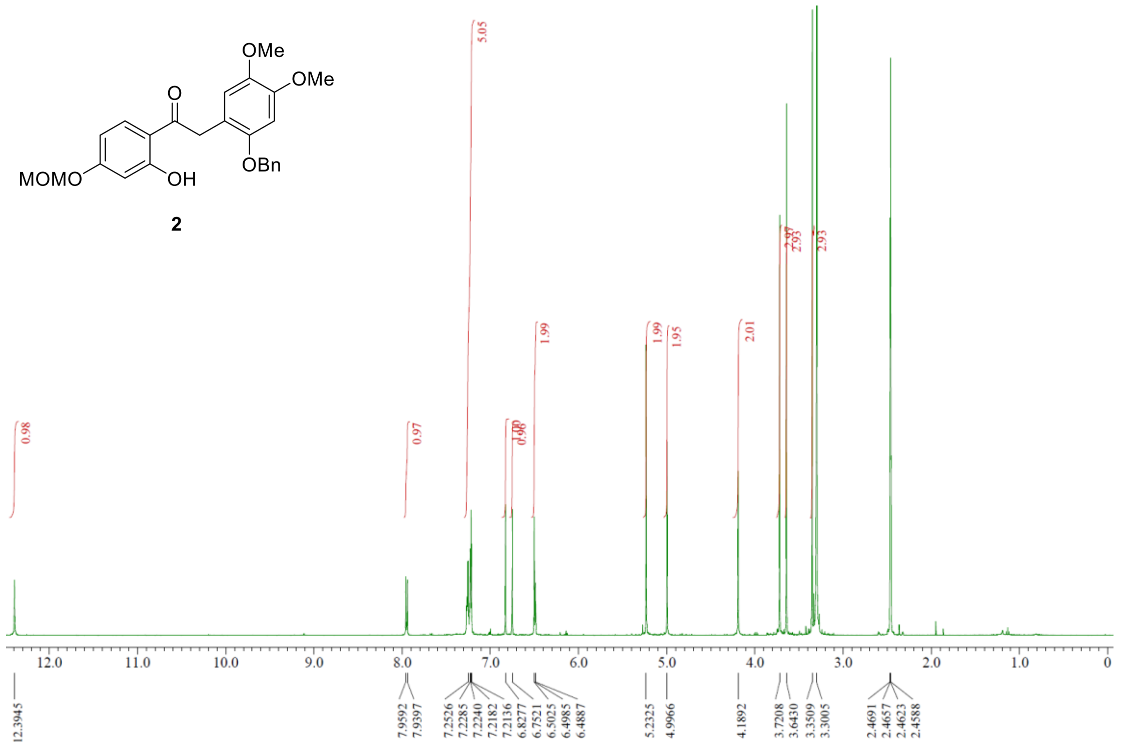

^{13}C NMR ($CDCl_3$, 125 MHz)

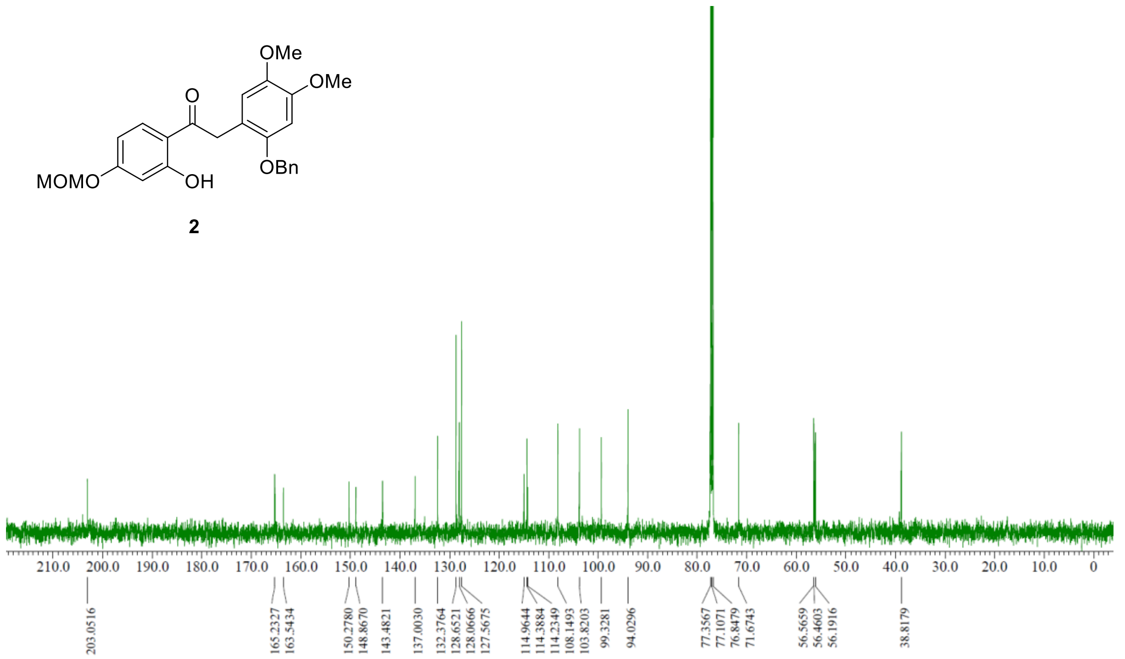

3-(2-(Benzyloxy)-4,5-dimethoxyphenyl)-7-(methoxymethoxy)chroman-4-one (**11**)

^{1}H NMR ($CDCl_3$, 500 MHz)

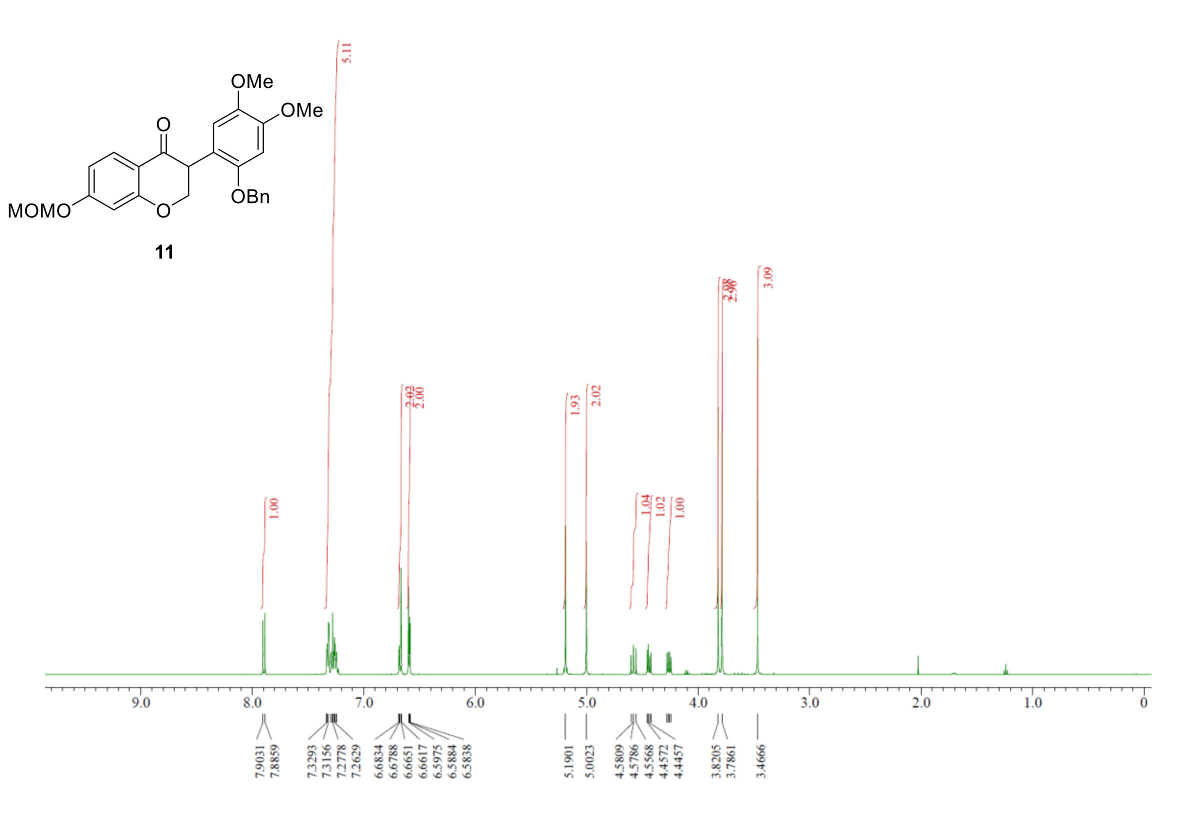

^{13}C NMR ($CDCl_3$, 125 MHz)

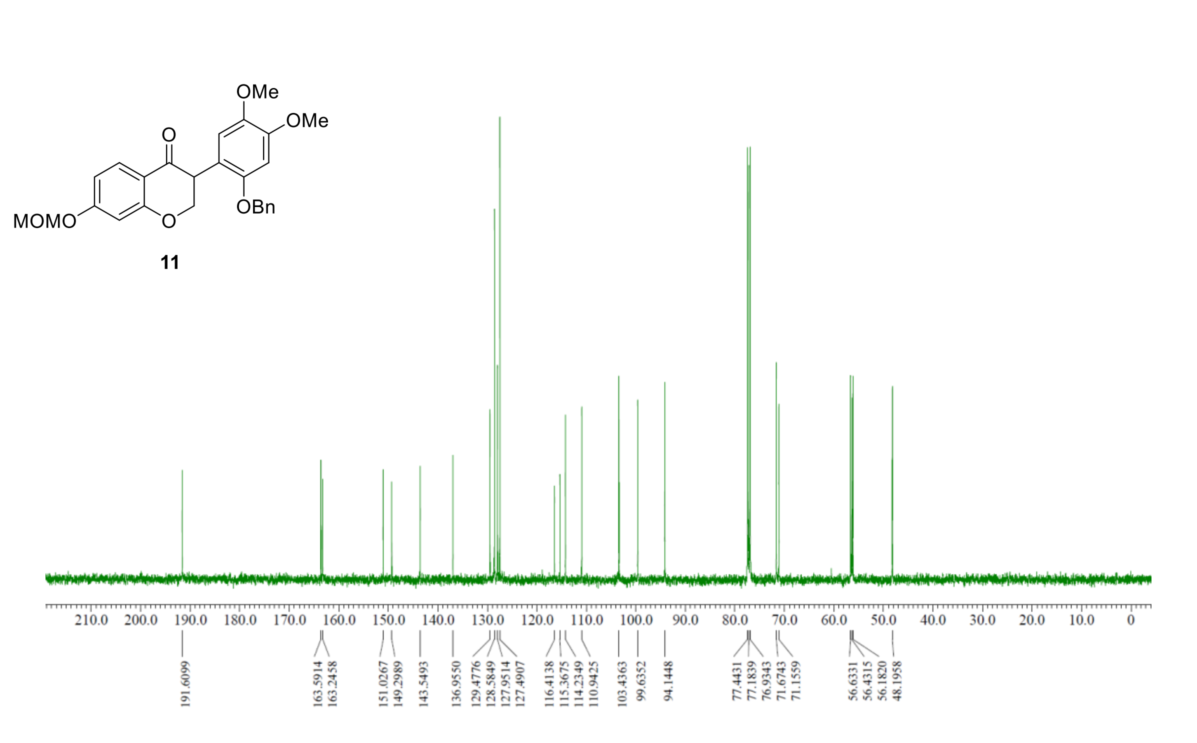

3-(2-(Benzyloxy)-4,5-dimethoxyphenyl)-7-hydroxychroman-4-one (**13**)

^{1}H NMR (DMSO-d$_6$, 500 MHz)

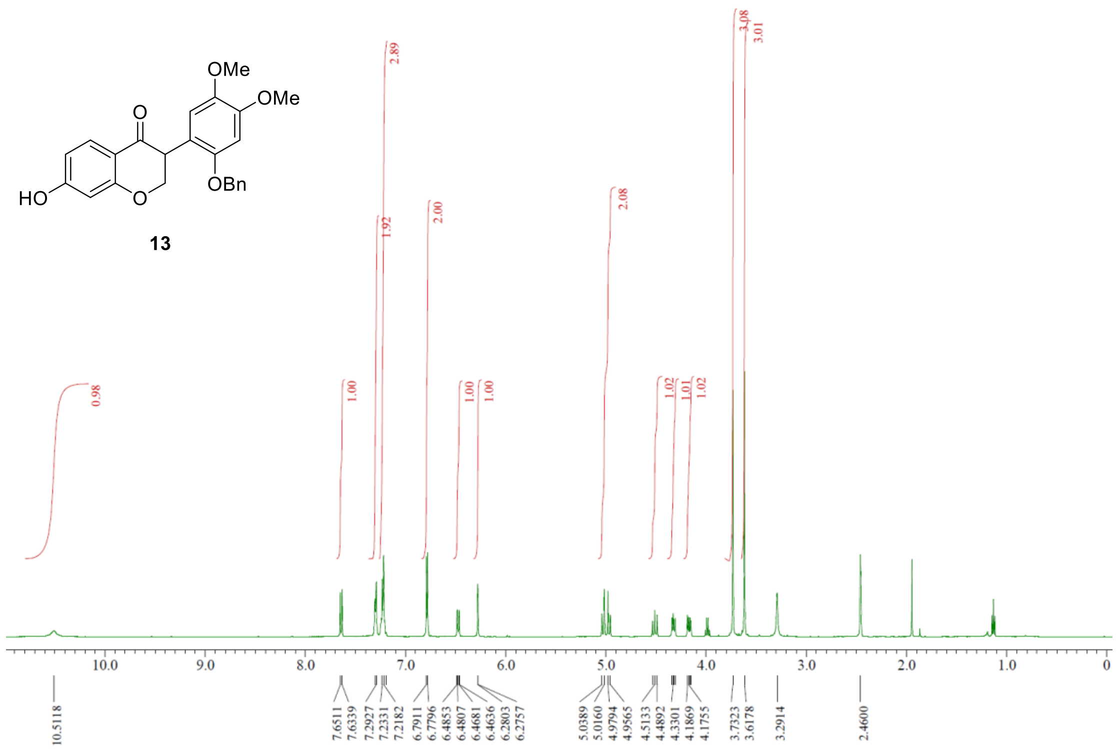

^{13}C NMR (DMSO-d$_6$, 125 MHz)

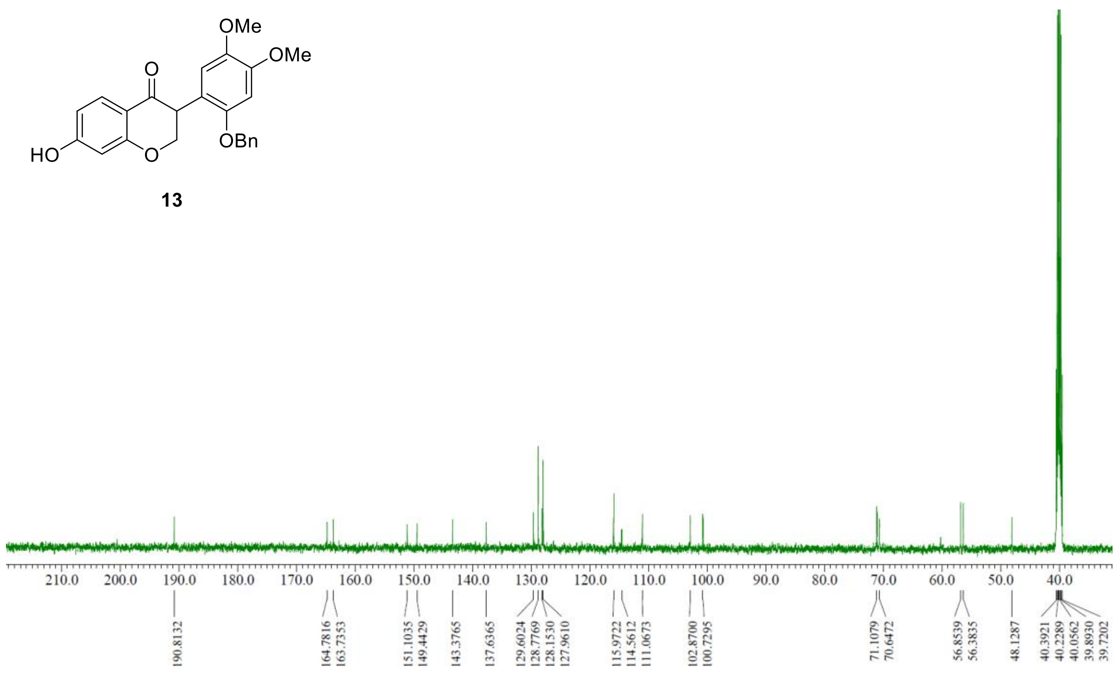

7,2`-Dihydroxy-4`,5`-dimethoxyisoflavanone (**1**)

^{1}H NMR (DMSO-d$_6$, 500 MHz)

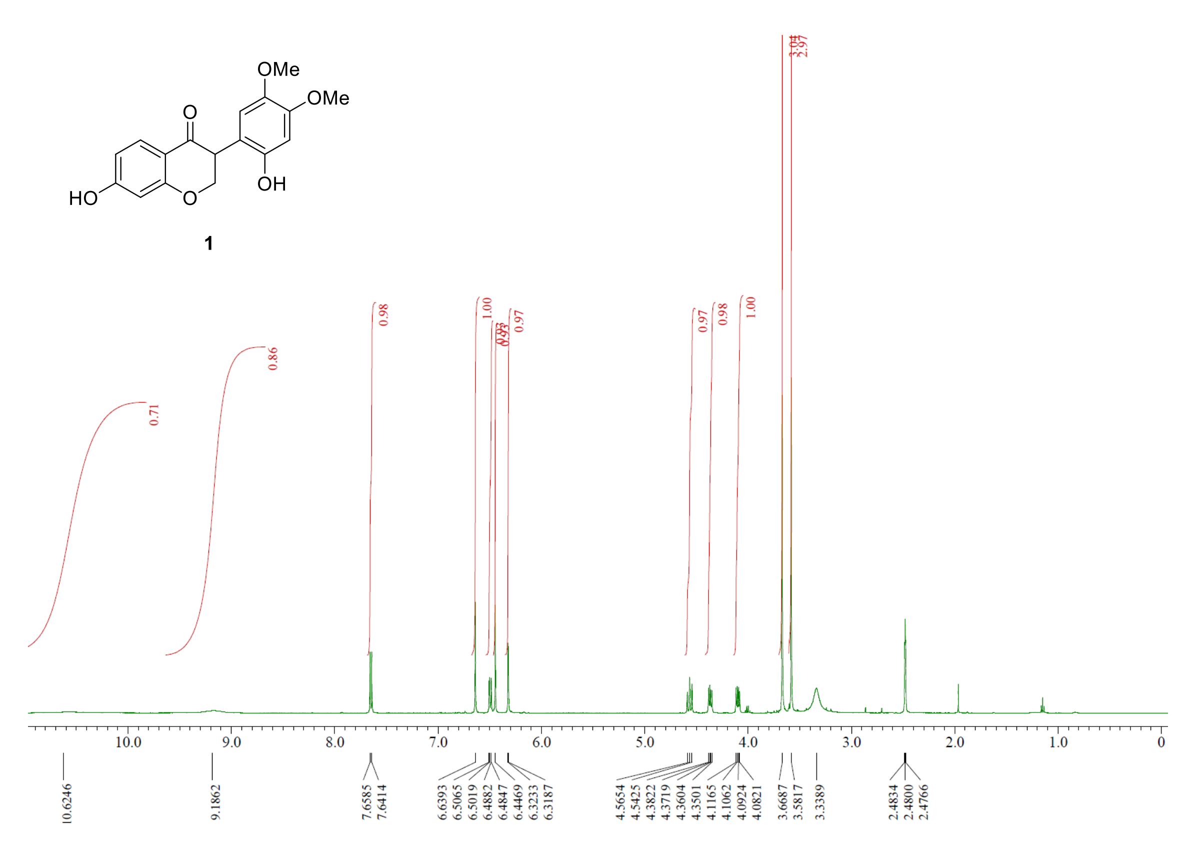

^{13}C NMR (DMSO-d$_6$, 125 MHz)

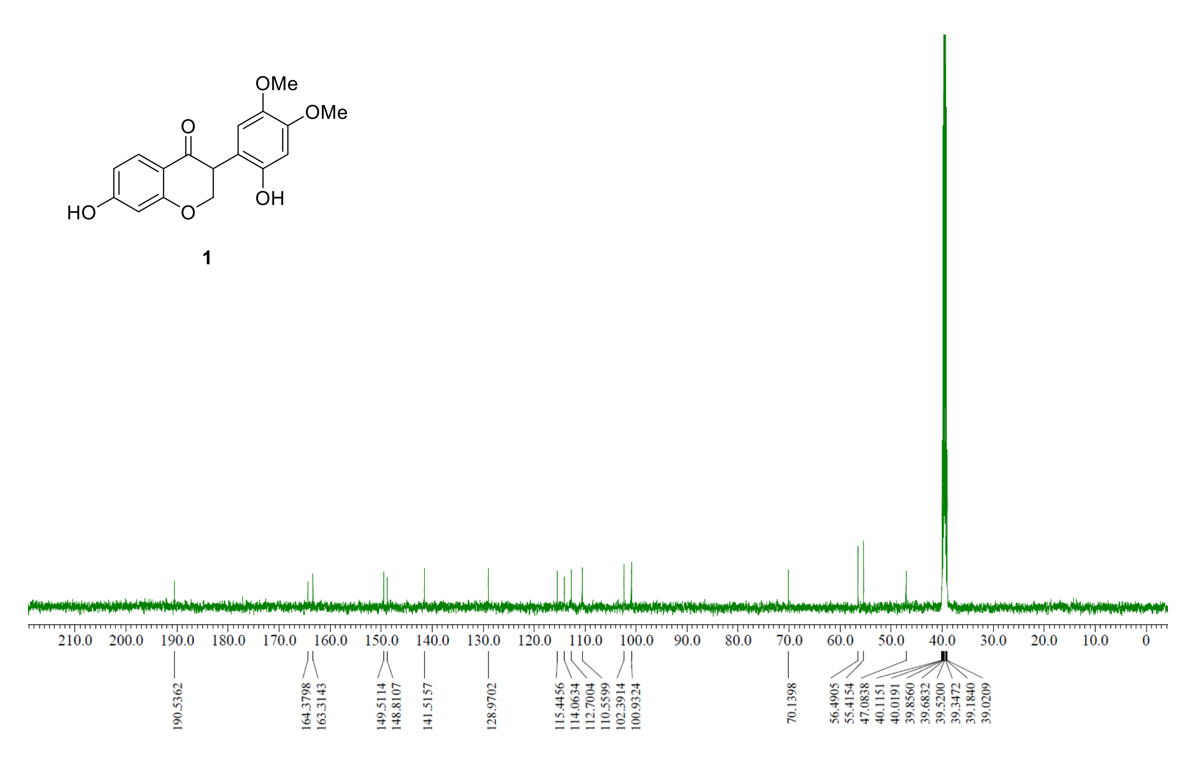

III. Comparison of Synthetic methods of 1

	Current method	Previous method (Kim et al.)[2]
Published journal	*Molecules* (2022)	*Bull. Korean Chem. Soc.* (2018)
Number of steps	11 steps<br>(from 3,4-dimethoxyphenol)	9 steps[2,3]<br>(from 3-benzyloxybenzaldehyde)[2]
Total yield	11%	> 12%[2,3]
Synthetic amounts of **1**	1.12 g	21 mg[2,3]

Table S2. Comparison of the synthetic features of current synthetic method for **1** with that of previous one.

IV . References

1. Beldjoudi, N., Mambu, L., Labaïed, M., Grellier, P., Ramanitrahasimbola, D., Rasoanaivo, P., Martin, M.T. and Frappier, F. Flavonoids from Dalbergia louvelii and Their Antiplasmodial Activity. *J. Nat. Prod*. **2003**, *66*, 1447-1450.

2. Singh, D.K., Kim, J., Sung, J.H. and Kim, I., 2018. Total Syntheses of Biologically Active Pterocarpan, Isoflavan, and Isoflavanone from Dalbergia oliveri. *Bull. Korean Chem. Soc.* **2018**, *39*, 239-243.

3. Pôças, E.S., Lopes, D.V., da Silva, A.J., Pimenta, P.H., Leitao, F.B., Netto, C.D., Buarque, C.D., Brito, F.V., Costa, P.R. and Noël, F., 2006. Structure–activity relationship of wedelolactone analogues: structural requirements for inhibition of Na+, K+-ATPase and binding to the central benzodiazepine receptor. *Bioorg. Med. Chem.* **2006**, *14*, 7962-7966.
